# New Derivatives of 2-(Cyclohexylamino)thiazol-4(5*H*)-one as Strong Inhibitors of 11β-Hydroxysteroid Dehydrogenase Type 1: Synthesis, Antiproliferative and Redox-Modulating Activity

**DOI:** 10.3390/ijms26188972

**Published:** 2025-09-15

**Authors:** Szymon Baumgart, Daria Kupczyk, Anita Płazińska, Oliwia Koszła, Przemysław Sołek, Aneta Archała, Wojciech Płaziński, Renata Studzińska

**Affiliations:** 1 Department of Organic Chemistry, Faculty of Pharmacy, Collegium Medicum in Bydgoszcz, Nicolaus Copernicus University in Toruń, 2 Jurasza Str., 85-089 Bydgoszcz, Poland; rstud@cm.umk.pl; 2 Department of Medical Biology and Biochemistry, Faculty of Medicine, Collegium Medicum in Bydgoszcz, Nicolaus Copernicus University in Toruń, 24 Karłowicza Str., 85-092 Bydgoszcz, Poland; dariak@cm.umk.pl; 3 Department of Biopharmacy, Medical University of Lublin, 4a Chodźki Str., 20-093 Lublin, Poland; anita.plazinska@umlub.pl (A.P.); koszlaoliwia@gmail.com (O.K.); pp.solek@gmail.com (P.S.); aneta.banach94@o2.pl (A.A.); wojtek_plazinski@tlen.pl (W.P.); 4 Department of Biochemistry and Toxicology, University of Life Sciences, 13 Akademicka Str., 20-950 Lublin, Poland; 5 Jerzy Haber Institute of Catalysis and Surface Chemistry, Polish Academy of Sciences, 8 Niezapominajek Str., 30-239 Krakow, Poland

**Keywords:** 11β-hydroxysteroid dehydrogenase 1, 2-aminothiazol-4(5*H*)-one derivatives, cortisol, molecular docking, cancer cell metabolic activity

## Abstract

In the present study, we synthesized nine new derivatives of 2-(cyclohexylamino)thiazol-4(5*H*)-one and evaluated their inhibitory activity against 11β-hydroxysteroid dehydrogenase type 1 and 2 (11β-HSD1 and 11β-HSD2), an enzyme responsible for the progression of metabolic disorders and cancers. All obtained derivatives showed inhibitory potential against 11β-HSD1, and four of them highly inhibited 11β-HSD1 activity with IC_50_ values in the low micromolar range. The most active compound, **3h** with IC_50_ = 0.04 µM, became a more potent and selective inhibitor than carbenoxolone. In addition to inhibition of 11β-HSD1, we investigated the antitumor potential and effects on intracellular redox homeostasis of all newly synthesized compounds on five cancer cell lines, namely human colon cancer (Caco-2), human pancreatic cancer (PANC-1), human glioma (U-118 MG), human breast cancer (MDA-MB-231), and skin melanoma (SK-MEL-30) and on healthy fibroblasts derived from the skin of a male neonate (BJ). Among the derivatives, all tested compounds were found to cause a decrease in cell viability for the MDA-MB-231 and Caco-2 lines and for compounds **3b**–**3i** for SK-MEL-30. The redox-modulating activity was assessed by measuring the levels of reactive oxygen species (ROS), reactive nitrogen species (RNS), and reduced glutathione (GSH) using the same panel of cancer lines and normal cells. This study showed an increase in ROS levels for SK-MEL-30, Caco-2, and MDA-MB-231 lines, while in the case of GSH levels, its reduction was observed in most experimental sets. The presented data suggest that the tested compounds are promising therapeutic agents with dual action because they offer the possibility of simultaneous regulation of metabolic disorders by inhibiting 11β-HSD1 and play a key role in anticancer therapy, which makes them prospective candidates for further clinical studies.

## 1. Introduction

11β-hydroxysteroid dehydrogenase type 1 (11β-HSD1) is an NAPH-dependent oxidoreductase, which in humans is mainly expressed in the liver, adipose tissue, lungs, central nervous system (CNS), and ovaries [1]. This enzyme, in the presence of the co-substrate nicotinamide adenine dinucleotide phosphate (NADPH), catalyzes the conversion of biologically inactive cortisone into biologically active cortisol [2]. Together with its second isoform, 11β-hydroxysteroid dehydrogenase type 2 (11β-HSD2), which catalyzes the reverse reaction, it maintains the balance of cortisol in the body. Through the participation of this enzyme in the regulation of cortisol levels, excessive expression of 11β-HSD1 in tissues contributes to the pathogenesis of, among others, Alzheimer’s disease (AD), depressive disorders, glaucoma, osteoporosis, cardiovascular diseases, or metabolic diseases such as obesity and type 2 diabetes [3,4,5,6,7,8]. In recent years, there has been increasing interest in the role of 11β-HSD1 in carcinogenesis. It has been shown that tumors can locally produce cortisol via 11-hydroxyteroid dehydrogenase type 1 by recycling circulating metabolites [9]. Locally elevated cortisol levels increase immunosuppression, which leads to an increase in the presence of regulatory T cells (Tregs). An increased number of Treg lymphocytes inhibits the activity of T helper lymphocytes (Th) and thus impairs the effective elimination of cancer cells and promotes tumor progression [10]. It has been shown that inhibition of 11β-HSD1 in mice with B16 melanoma tumors led to regression of tumor growth, comparable to that obtained in mice with genetically deleted 11β-HSD1 [9]. 11β-HSD1 activity has also been associated with reduced response to immune checkpoint inhibitor (ICI) treatment in non-small cell lung cancer, renal cell carcinoma, and melanoma [11,12,13]. Dysregulated cortisol metabolism in the tumor microenvironment, which is caused by, among others, overactivity of 11β-HSD1, inhibits the functioning of immune system cells and thus reduces the effectiveness of ICI. The use of small molecule 11β-HSD1 inhibitors in combination with a PD-1 inhibitor in a mouse melanoma model led to better control of tumor growth than using PD-1 blockade alone [13]. Therefore, the combination of 11β-HSD1 inhibitors and immunotherapy represents a new potential therapeutic strategy for cancer.

**Figure 1:**
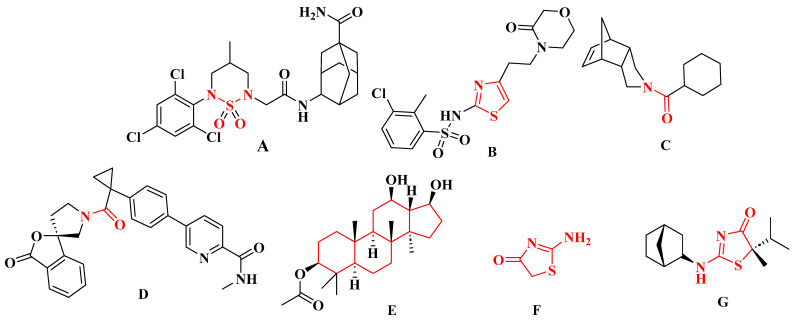
Structures of selected selective inhibitors of 11β-HSD1. (**A**) Compound with a sulfonamide moiety; (**B**) compound with a 2-aminothiazole scaffold; (**C**,**D**) compounds with an amide group; (**E**) compound based on the hexadecahydro-1*H*-cyclopenta[α]phenanthrene system; (**F**) compound with a 2-aminothiazol-4(5*H*)-one (pseudothiohydantoin) core; (**G**) AMG-221.

In light of recent reports on the role of 11β-HSD1 in the development of several types of cancer and the possibility of using 11β-HSD1 inhibitors in pharmacotherapy, the search for new compounds—selective 11β-HSD1 inhibitors—becomes interesting. Already-known 11β-HSD1 inhibitors (Figure 1) include compounds containing a sulfonamide moiety (A) [14], 2-aminothiazole (B) [15], amide (C, D) [16,17], or based on the hexadecahydro-1*H*-cyclopenta[α]phenanthrene system (E) [18]. Attractive compounds that have aroused our interest are those with a 2-aminothiazol-4(5*H*)-one (pseudothiohydantoin) core (F). This group of compounds includes compounds with high inhibitory activity, and therefore, some representatives of this group have been subjected to clinical evaluation, among others, a selective 11β-HSD1 inhibitor from Amgen and Biovitrum—AMG-221 (G) (however, research on this compound was discontinued) [19]. Therefore, taking into account the information that the 2-aminothiazol-4(5*H*)-one moiety is a promising scaffold for drug design, in the course of our several-year research on the search for selective 11β-HSD1 inhibitors, we mainly modified the substituents on the amino group of 2-aminothiazol-4(5*H*)-one to obtain stronger and more selective 11β-HSD1 inhibitors [20,21,22]. These studies resulted in the conclusion that the presence of large hydrophobic groups at the amino group leads to an increase in inhibitory activity.

The aim of this study was, first, to design and synthesize new compounds containing the 2-(cyclohexylamino)thiazol-4(5*H*)-one system and to evaluate their selective inhibitory activity against 11β-hydroxysteroid dehydrogenase type 1. The selection of 2-(cyclohexylamino)thiazol-4(5*H*)-one derivatives was justified by the results of studies based on the developed quantitative structure-activity relationship (QSAR) model, aimed at searching for new 11β-HSD1 inhibitors [23]. QSAR analysis showed that cyclohexyl derivatives constitute a promising group of compounds intended for further synthesis and biological evaluation. The obtained compounds were tested against human colon cancer (Caco-2), breast cancer (MDA-MB-231), melanoma (SK-MEL-30), glioma (U-118 MG), and pancreatic cancer (PANC-1) cell lines using the 3-(4,5-dimethylthiazol-2-yl)-5-(3-carboxymethoxyphenyl)-2-(4-sulfophenyl)-2*H*-tetrazolium (MTS) assay. Additionally, in the end, the level of reactive oxygen and nitrogen species (ROS/RNS) was examined in all six tested cell lines.

## 2. Results and Discussion

### 2.1. Chemistry

**Figure 2:**
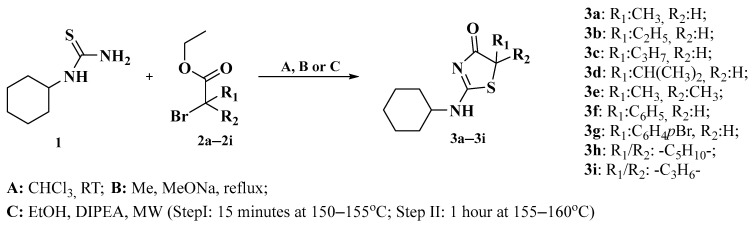
Synthesis of 2-aminothiazol-4(5*H*)-one derivatives **3a**–**3i**.

In this work, all new 2-(cyclohexylamino)thiazol-4(5*H*)-one derivatives were synthesized according to the reaction shown in Figure 2. The synthesis of *N*-cyclohexyl substituted 2-aminothiazol-4(5*H*)-one derivatives, depending on the bromo ester used, was carried out using three different methods (for a description of the methods, see Section 3.2).

Method A, similarly to previously conducted syntheses of pseudothiohydantoin derivatives [20], was effective in synthesizing derivatives **3a**–**3c**. The reaction carried out according to method A allowed the obtaining of compounds **3a**–**3c** in the form of hydrobromides with high yield (54.79–82.75%). The synthesis of compounds **3d** and **3e** by method A turned out to be ineffective because no reaction products appeared on TLC plates after both reactions were carried out for 14 days. Therefore, an attempt was made to synthesize **3d**–**e** derivatives using method B. This method allowed the obtainment of **3d**–**e** derivatives with a yield of 11.78–14.55%. However, based on method C, thanks to which we had previously successfully obtained analogously compounds in the 2-(cyclopentylamino)thiazol-4(5*H*)-one series, compounds **3f**–**3i** were obtained (with yields in the range of 1.40–74.68%). In the case of compound **3i**, the low yield is related to losses when isolating the compound from the post-reaction mixture using column chromatography. The structures of all nine new derivatives were confirmed using ^1^H NMR, ^13^C NMR, and HRMS. Yield and melting point data are shown in Table 1.

### 2.2. 11β-HSD Inhibitory Activity

The obtained compounds **3a**–**3i** were tested in vitro for their inhibitory activity against 11β-HSD1 and 11β-HSD2. The test results are summarized in Table 2. In seven of the nine 2-(cyclohexylamino)thiazol-4(5*H*)-one derivatives, over 80% inhibition of 11β-HSD1 was observed, while in the 2-(cyclopentylamino)thiazol-4(5*H*)-one derivatives, only three compounds showed such inhibitory potency [20]. This indicates that the introduction of a larger hydrophobic substituent at the amino group of pseudothiohydanotoin leads to an increase in the potency of 11β-HSD1 inhibitors.

Moreover, overall, as a series of derivatives, they have so far shown the highest inhibitory activity among all 2-aminothiazol-4(5*H*)-one derivatives synthesized by our research group. In the series of 2-(*tert*-butylamino)thiazol-4(5*H*)-one [22] and 2-(adamantylamino)thiazol-4(5*H*)-one [21] derivatives, only one compound out of nine exceeded 80% of the inhibitory activity against 11β-HSD1 at a concentration of 10 µM. However, in the case of the series of 2-(methylamino)thiazol-4(5*H*)-one [24], 2-(isopropylamino)thiazol-4(5*H*)-one [25], and 2-(allylamino)thiazol-4(5*H*)-one [26], no compound reached such a level of inhibition.

Among the tested compounds, the best inhibitory effect on 11β-HSD1 was demonstrated by derivative **3h**, with a value of IC_50_ = 0.04 µM. This compound inhibited the activity of 11β-HSD1 more strongly than carbenoxolone used as a control (IC_50_ = 0.08 µM). Derivative **3i** was also characterized by strong inhibition of 11β-HSD1 with an IC_50_ value of 0.09 µM. The activity of the **3i** derivative is almost twice as low as the activity of the **3h** derivative. This indicates that the presence of a smaller ring in the spiro system with the thiazole ring influences the decrease in inhibitory activity against 11β-HSD1. The activity tests also showed that the derivative containing a 4-bromophenyl group in the fifth position of the thiazole ring (compound **3g**) is characterized by higher activity than derivative 3f, which contains a phenyl group. The IC_50_ values of these compounds are 0.07 µM (**3g**) and 0.17 µM (**3f**), respectively.

Among the derivatives with branched aliphatic substituents, derivative **3d** was the most potent inhibitor (IC_50_ = 0.08 µM). Derivative **3e** also demonstrated good activity with an IC_50_ of 0.3 µM.

The highest activity among the unbranched derivatives was obtained for 2-(cyclohexylamino)-5-propylthiazol-4(5*H*)-one (**3c**), with an IC_50_ of 0.4 µM. Introducing an ethyl group at the 5-position of the thiazole ring in compound **3b** resulted in a decrease in 11β-HSD1 inhibition (IC_50_ for compound **3b** is 0.57 µM). A significant decrease in activity occurred after the introduction of the smallest methyl substituent (**3a**)—the percentage of 11β-HSD1 inhibition at a concentration of 10 µM was minimal and amounted to 27.48% (IC_50_ > 10 µM). The obtained results indicate that increasing the length of the straight aliphatic chain leads to a gradual increase in enzyme inhibition, suggesting that a longer alkyl substituent likely promotes an increase in ligand-enzyme binding affinity.

However, tests for the inhibition of 11β-hydroxysteroid dehydrogenase type 2 showed that all obtained derivatives of 2-(cyclohexylamino)thiazol-4(5*H*)-one at a concentration of 10 µM inhibited 11β-HSD2 in the range from 38.34% to 50.97%. Compound **3i** was the most active against 11β-HSD2 (at a concentration of 10 µM, it showed 50.97% inhibition of 11β-HSD2). In the case of only one derivative, derivative **3a**, greater inhibitory activity towards 11β-HSD2 than 11β-HSD1 was observed.

Considering the obtained in vitro test results in terms of 11β-HSD1 inhibition potency and selectivity, compounds **3d**, **3g** and **3h** should be distinguished because they are characterized by strong inhibitory activity and greater selectivity than the standard compound—carbenoxolone.

### 2.3. Results of the Docking Studies

**Figure 3:**
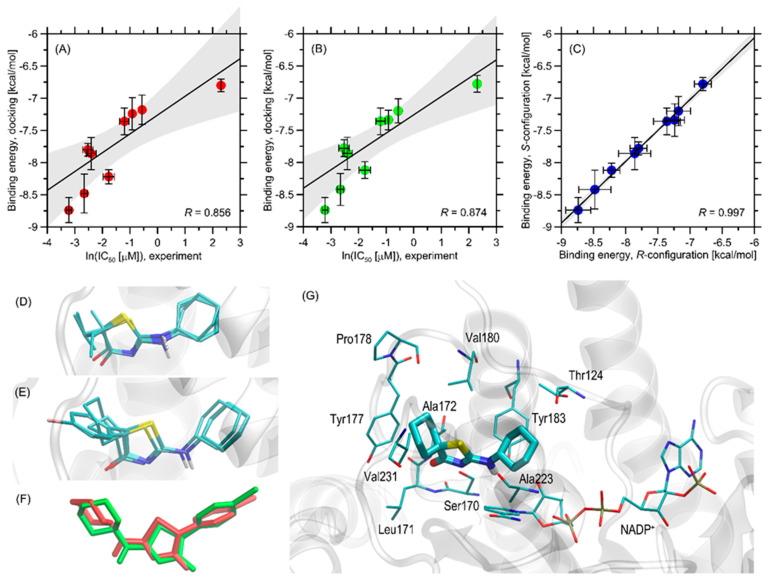
(**A**) The correlation between the binding energies calculated for compounds with R-configuration at the chiral center, averaged across the five protein structures used for docking, and their corresponding (experimental) IC_50_ values (recalculated as log(IC_50_)). The horizontal bars indicate the standard deviation within the dataset. The solid line shows the linear regression, with 95% confidence intervals shaded in grey. (**B**) Similar to (**A**), but for compounds with *S*-configuration. (**C**) The correlation of theoretical binding energies for compounds with *R*- and *S*-configurations. (**D**) The superposed optimal positions of all ligands **3a**–**3e** with *R*-configuration (stick representation) in the binding cavity of the PDB:4bb5 structure. (**E**) Similar to (**D**), but for compounds **3f**–**3i**. (**F**) The superposition of the two protein-bound stereoisomers of compound **3g**. (**G**) The energetically favorable location of the **3h** compound molecule bound to the PDB:4bb5 structure. The ligand molecule is shown with thick sticks, while the closest amino-acid residues (<0.4 nm) are shown with thin sticks. The NADP^+^ molecule present in the protein crystal structure is also included. Descriptions of interaction types are provided in the text.

The binding energies from the docking simulations are shown in Figure 3A–C. The determined binding energies for the ligands fall within a narrow range of approximately −8.8 to −6.8 kcal/mol, indicating favorable inhibitor-protein interactions. These energies are consistently more favorable compared to a previously studied group of compounds [20]. For structurally similar compounds differing only by a single substituent (cyclohexyl vs. cyclopentyl group), the average difference in binding energy is around 0.34 kcal/mol. The effect of stereoselectivity on binding strength is minimal, with binding energy differences between stereoisomers not exceeding 0.06 kcal/mol. Additionally, there is a high correlation between the theoretical binding energies for compounds with either *R*- or *S*-configuration (R = 0.997, Figure 3C), indicating that the stereoconfiguration of chiral ligands does not significantly impact binding affinity. This is supported by the similar orientation of protein-bound stereoisomers of the same ligand, as exemplified by compound **3g** in Figure 3C.

The order of theoretically determined binding energies accurately reflects the experimental IC_50_ values for those compounds with measured IC_50_ (Figure 3A,B), correctly identifying the most potent (**3h**) and least potent (**3a**) compounds. For the entire set of compounds, there is a clear correlation between ln(IC_50_) and binding energy, with correlation coefficients of 0.856 for *R*-configuration compounds (*p* = 0.00327) and 0.874 for *S*-configuration compounds (*p* = 0.00207). The potency trend aligns with the variations in theoretical binding energies for all compounds except **3d** and **3i**. For the least potent compound, **3a**, with an undetermined but high IC_50_ value (>10 μM), an IC_50_ of 10 μM was assumed.

Given the satisfactory agreement between theoretical and experimental results, we conducted a more detailed analysis to identify the structural aspects of ligand-protein interactions. The summary below is based on analyzing ligand-protein contacts where the distance between any corresponding atom pair is smaller than an arbitrarily accepted value of 0.4 nm.

All the studied ligands bind to the protein structure in a nearly identical manner (see Figure 3D), and their orientation in the binding cavity closely resembles that of a structurally related group of ligands from our previous study [26]. There is a particularly strong similarity between the currently studied compounds and the group from ref. [19], which differ only by one substituent (cyclohexyl instead of the cyclopentyl group). Considering the binding energy data, it can be concluded that exchanging this group results in an additive contribution to the binding energy and a systematic increase in ligand-protein affinity. Alternative poses, not included in Figure 3D,E, are associated with significantly higher energy levels (at least 0.6 kcal/mol above the most favorable energy level) and do not form structurally consistent clusters; thus, they were excluded from the analysis. The similarity in docking poses also extends to the stereoisomers of the same compound (Figure 3F). However, it should be noted that the ligand orientation is slightly dependent on the substituent size on the thiazole ring; compounds **3a**–**3e** and **3f**–**3i** form slightly different clusters with minor reorientation of the thiazole ring substituents (Figure 3D,E) due to the steric hindrance of the bulkier cyclohexyl ring with neighboring amino acid residues.

The detailed description of the protein-ligand contact pattern focuses on the most potent compound **3h**. However, the interaction pattern found is representative of all studied compounds that exhibit inhibition properties. The graphical illustration of the docking results is provided in Figure 3G.

The aliphatic cyclohexyl group present in all studied ligands maintains close contacts with the side chains of Tyr183 and Thr124. These interactions are primarily CH–π interactions and hydrophobic contacts involving only the non-polar parts of the Tyr and Thr sidechains. Similar non-polar interactions occur between the cyclohexyl rings and Val180, although these molecular fragments are slightly farther apart compared to the interactions with Tyr183 and Thr124. The proximity of the NADP^+^ molecule, especially its polar fragments, appears to be an opportunistic outcome of these interactions. The amine group of the ligand, located centrally, interacts with the amide group of NADP^+^ and Ser170. Although the distance between potential donors and acceptors is too great for stable hydrogen bonds, such attractive contacts might occur when considering conformational heterogeneity driven by temperature and the presence of a solvent. The thiazole ring in the central part of the ligand interacts with Ser170 (via sidechain hydrogen bonding) and Ala172 (via backbone hydrogen bonding). The aromatic residue of NADP^+^ is too distant for stable edge-to-edge π–π stacking, but minor energy contributions from this type of interaction are likely. Finally, the various substituents differing across the considered set of compounds and attached to the thiazole ring make contact with several non-polar amino acid residues, forming a hydrophobic core that includes Leu171, Leu217, Tyr177, and Ala172. Val180, located at the edge of this cluster, is closer to the opposite side of the ligand molecule and the thiazole ring, but also supports this network of non-polar interactions. The contacts with Tyr177 are CH–π interactions, while those with leucines, Ala172, and possibly Val180 are hydrophobic interactions that contribute to binding energy by minimizing the solvent-exposed non-polar surfaces.

In summary, the primary driving force for binding appears to be the hydrophobic interactions between the ligand and the non-polar cluster (Leu171, Leu217, Tyr177, and Tyr183), supported by hydrogen bonding with Ser170 and possibly NADP^+^. The variations in binding energies across the group of compounds can be attributed to interactions between the substituents on the thiazole ring and the region of the non-polar cluster. Finally, in comparison to a previously studied group of compounds [20], the currently observed enhanced affinity is a consequence of the increased size of the cyclohexyl substituent, which enables more favorable CH–π interactions with Tyr183.

### 2.4. Metabolic Activity

**Figure 4:**
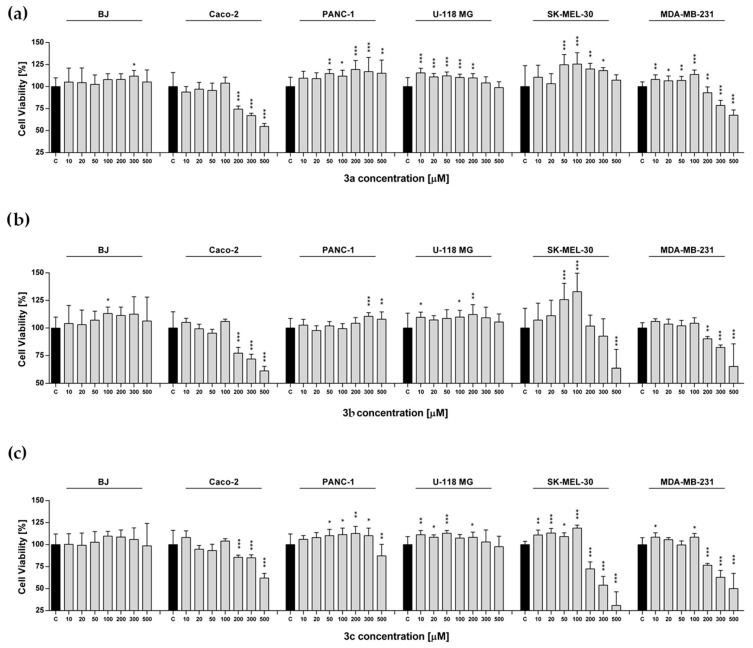

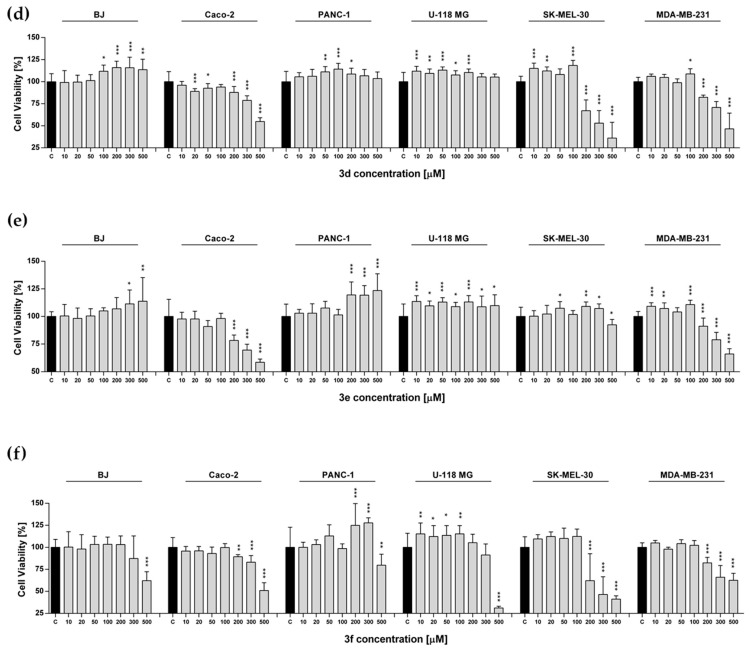

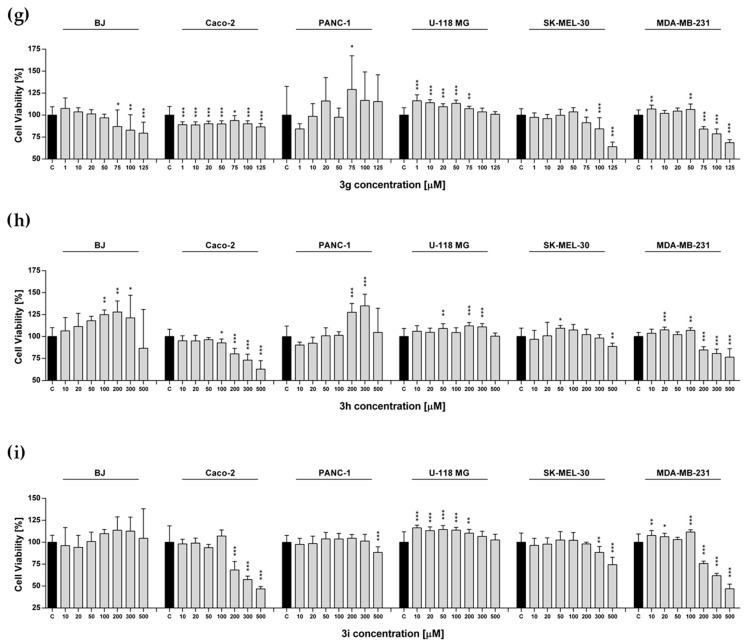
(**a**–**i**)**.** Observed differences in metabolic activity in cancer and normal (BJ) cell lines exposed to compounds **3a**–**3i** over a wide range of concentrations for 72 h. Cell viability was assessed using the MTS assay. Bars in the graphs represent the mean value and standard deviation, n = 8, *** *p* < 0.001, ** *p* < 0.01, * *p* < 0.05 (one-way analysis of variance and Dunnett’s a posteriori test).

We observed significant changes in cell metabolic activity in a concentration-dependent manner. In detail, we noted that the metabolic activity of human fibroblast, human brain glioma, and human pancreatic cancer cells increased or remained unaffected except for compound **3f** for the U-118 MG cells, compounds **3f** and **3g** for the BJ cells, and compounds **3c**, **3f**, and **3i** for PANC-1 (Figure 4). The highest increase in proliferation was observed for compounds **3e**, **3f**, and **3h** for PANC-1 and **3a**, **3g**, and **3i** for U-118 MG; it corresponded to the range of 115–134% (Figure 4). In contrast, we noted a decrease in cell viability for the MDA-MB-231, Caco-2, and SK-MEL-30 lines treated with all tested substances except for **3a** and SK-MEL-30. More precisely, compounds **3b**, **3e**, **3g**, **3h**, and **3i** for Caco-2, **3b**, **3c**, **3g**, **3h**, and **3i** for MDA-MB-231, and **3c**, **3d**, **3f**, and **3g** for SK-MEL-30 exhibited the strongest effects and resulted in cell metabolic activity down-regulation to 30–54% (Figure 4). Our findings suggest that the tested compounds, analogs of 2-(cyclohexylamino)thiazol-4(5*H*)-one, may exhibit anticancer effects by reducing cellular metabolic activity. This is consistent with other studies performed for 2-(cyclopentylamino)thiazol-4(5*H*)-one derivatives, which have shown inhibition of tumor progression through metabolic suppression [20]. The biological activity of the tested compounds results from the presence of the 2-aminothiazole group, which has not only enzyme-inhibiting properties, but also anticancer properties [20,21,22,27].

The decrease in metabolic activity of the tested MDA-MB-231 and SK-MEL-30 cell lines observed for derivatives with cyclopentylamine and cyclohexylamine groups is at a similar level, and a significant reduction in cell viability is observed mainly at higher concentrations of the tested compounds, above 200 µM. The previous research on the Caco-2 cell viability for 2-(cyclopentylamino)thiazol-4(5*H*)-one derivatives showed that compounds **3a**–**3e** and **3i** exhibit the strongest activity at concentrations above 10–20 µM. Replacing the cyclopentylamine group with a cyclohexylamine group in the molecular structure of the tested compounds resulted in a decrease in Caco-2 cell viability at concentrations 10–20 times higher, above 200 µM (compounds **3a**, **3b**, **3d**, **3e**, and **3i**). For compound **3c**, a fifty-fold lower activity towards Caco-2 is observed. However, for compound **3g** from the group of 2-(cyclohexylamino)thiazol-4(5*H*)-one derivatives, we observed the strongest effect of decreasing cell viability for the Caco-2 line at a concentration of 1 μM. Only for compound **3g**, replacing the cyclopentyl group with a cyclohexyl group leads to a two-hundred-fold reduction in concentration, resulting in a statistically significant decrease in metabolic activity for the Caco-2 cell line. It is important to emphasize that compound **3g** was the only one among the tested 2-(cyclohexylamino)thiazol and 2-(cyclopentylamino)thiazol derivatives to cause a reduction in the metabolic activity of breast cancer cells at concentrations of 75 µM and 50 µM, respectively. Due to this remark, compound **3g** may constitute a pharmacophore for subsequent derivatives in the search for a compound for the treatment of colorectal cancer.

In the case of PANC-1 and U-118 MG cell lines, for the derivatives of 2-(cyclopentylamino)thiazol-4(5*H*)-one and 2-(cyclohexylamino)thiazol-4(5*H*)-one, the increase in the cell metabolic activity by approximately 115 to 144% was observed [20]. On this basis, it can be concluded that derivatives of 2-(cyclopentylamino)thiazol-4(5*H*)-one and 2-cyclohexylamino)thiazol-4(5*H*)-one show a similar level and trend in anticancer activity towards the cell lines: PANC-1, U-118 MG, MDA-MB-231, and SK-MEL-30; significant differences concern only the effect on Caco-2.

### 2.5. Evaluation of Intracellular Redox Homeostasis

**Figure 5:**
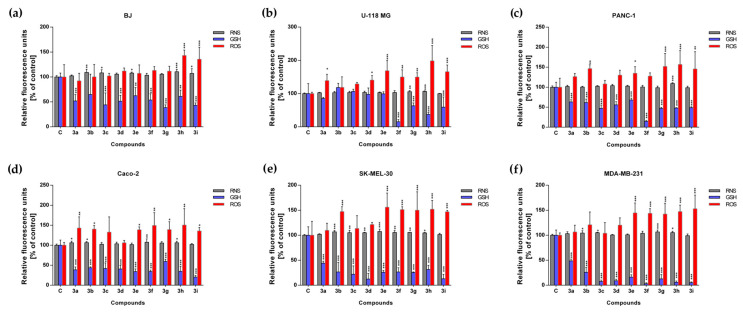
Redox state of cancer and normal cells: (**a**) BJ, (**b**) U-118-MG, (**c**) PANC-1, (**d**) Caco-2, (**e**) SK-MEL-30, (**f**) MDA-MB-231, after 72 h of exposure to compounds **3a**–**3i**. Bars presented in the graphs represent the mean value and standard deviation, n = 8, *** *p* < 0.001, ** *p* < 0.01, * *p* < 0.05 (one-way analysis of variance and Dunnett’s a posteriori test).

The levels of reactive oxygen species, reactive nitrogen species, and reduced glutathione (GSH) were measured for all experimental sets at a concentration of 500 µM/each. The highest increase in the level of the reactive oxygen species (ROS) was noted for the SK-MEL-30, Caco-2, and MDA-MB-231 lines. These findings demonstrate that the tested compounds exert pro-oxidant activity, a mechanism considered therapeutically advantageous in anticancer strategies. The associated increase in ROS production in these cell lines is likely to compromise cellular homeostasis and trigger apoptotic pathways. In detail, we observed an increase in ROS production for compounds **3a**, **3b**, **3e**–**3i** in the case of Caco-2, for **3b**, **3e**–**3i** in the case of SK-MEL-30 cells, and **3e**–**3i** in the case of MDA-MB-231. For the BJ line, we did not observe an increase in ROS, with the exception of compounds **3h** and **3i** (Figure 5).

In the case of RNS, we observed an increased level for the BJ line, with the exception of compounds **3a**, **3d**, **3f**, **3g**, and for SK-MEL-30 with the exception of **3a** and **3i**. We noted that the reactive nitrogen species (RNS) level for the U-118-MG, PANC-1, MDA-MB-231 and Caco-2 cell lines remained unaffected except for compounds **3g** and **3h** for U-118-MG, 3h for PANC-1, **3b**, **3c**, **3g** and **3h** for MDA-MB-231 as well as **3a**, **3b**, **3f** and **3h** for Caco-2 (Figure 5). Interestingly, the level of glutathione (GSH) was reduced in all experimental sets, except for compounds **3b** for the BJ cell line and **3a**–**3e** for U118-MG (Figure 5). A similar trend was observed for compounds **3a**–**3i** from the 2-(cyclopentylamino)thiazol-4(5*H*)-one derivatives [20].

GSH is a critical intracellular antioxidant that plays a role in a primary defense mechanism by regulating cellular redox homeostasis and protecting cells from oxidative and nitrosative stress caused by reactive oxygen and nitrogen species. The observed depletion of GSH is directly associated with the induction of pro-oxidant stress, as diminished GSH levels compromise the cellular antioxidant defense, thereby increasing the susceptibility of cancer cells to oxidative damage and impairing their capacity to detoxify ROS. This impairment represents a critical event in the initiation of cell death pathways and contributes to the antiproliferative activity of the tested compounds. As expected, glutathione activity increases in response to the presence of water-derived free radicals. Moreover, elevated intracellular GSH levels in cancer cells have been correlated with tumor progression and the development of resistance to chemotherapeutic agents. Similar to the cyclopentylamino derivatives, the strongest GSH reduction was observed for the MDA-MB-231 cell line. Replacing the cyclopentylamino group with the cyclohexylamino group resulted in a significant reduction in GSH levels. Our results provide evidence that cyclohexylamino derivatives exhibit enhanced pro-oxidant activity compared to their cyclopentylamino analogs, with the MDA-MB-231 cell line showing the greatest sensitivity to this effect, while for cyclopentylamino derivatives, the most significant reduction was observed for compounds **3f**–**3i**, mainly for the MDA-MB-231 line, and for cyclohexylamino derivatives, a reduction in the GSH level was observed for all compounds. Moreover, replacing the cyclopentyl group with a cyclohexyl group resulted in a decrease in the GSH concentration for Caco-2, except for compound **3g** [20].

### 2.6. Predicting Medicinal Chemistry Structural Alerts

In the early stages of drug discovery, a key step is to identify compounds that meet the parameters of medicinal chemistry. This study assessed pseudothiohydantoin derivatives for potential interferences in the assay using the PAINS (Pan-assay interference compounds) test and the Brenk alert. Compounds that interfere with the pan-assay test are structures that interfere with biological assays by giving false positive results [28]. The Brenk filter is used to identify compounds that contain undesirable functional groups in their structure, which may cause toxicity, metabolic instability, and poor pharmacokinetics [29]. SwissADME software was used to conduct this study [30]. The analysis showed that derivatives **3a**–**3i** did not show any of the alerts in either above-described filters used in medicinal chemistry (the Supplementary Information includes the outcomes analysis (see Table S1)). This means that the compounds are beneficial for further biological studies.

## 3. Materials and Methods

### 3.1. Chemistry

Microwave reactions were conducted using a MAGNUM V2 microwave reactor/mineralizer from ERTEC (Wrocław, Poland). The melting points given were determined in open capillaries (capillary uncorrected) using a Digi-Melt MPA160 melting point apparatus from SRS (Sunnyvale, CA, USA). ^1^H NMR and ^13^C NMR spectra were recorded on Bruker Avance 400 and 700 instruments (TMS as an internal standard, Bruker Billerica, MA, USA). High-resolution mass spectrometry (HRMS) measurements were performed using a Synapt G2 Si mass spectrometer (Waters, Warsaw, Poland). The measurement results were prepared in the MassLynx 4.1 program (Waters, Warsaw, Poland). Thin-layer chromatography (TLC) was performed using 5 × 10 cm plates coated with F254 silica gel (Merck, Darmstadt, Germany). Gravity column chromatography was performed on MN Kieselgel 60M silica gel with a grain diameter of 0.04–0.063 mm (Machery-Nagel, Oensingen, Switzerland).

The starting compounds, *N*-cyclohexylthiourea, were purchased from abcr GmbH (Karlsruhe, Germany), and the 2-bromoesters: ethyl 2-bromopropionate 99%, 2-bromobutyrate 98%, 2-bromovalerate 99%, 2-bromo-3-methylbutyrate 95%, 2-bromoisobutyrate 98%, 2-bromophenyl acetate 97%, 2-bromo(4-bromophenyl) acetate 97%, bromocyclobutane carboxylate 95% and methyl 1-bromocyclohexane carboxylate 97%, are widely available commercially (Alfa Aesar, Kandel, Germany, Acros Organic, Geel, Belgium, Sigma-Aldrich, Poznań, Poland). The remaining auxiliary reagents and solvents were purchased from commercial suppliers with a high degree of purity without the need for further purification.

### 3.2. Synthesis Procedures

#### 3.2.1. Method A

In a flask containing 0.002 mol of *N*-cyclohexylthiourea (**1**) and 0.0022 mol of the appropriate 2-bromoester (**2a**–**2c**), 27 mL of chloroform was added. The reaction mixture was stirred at room temperature for 14–21 days. The course of the reaction was monitored by thin-layer chromatography (TLC) (eluant: ethyl acetate). After completion of the reaction, the solvent was evaporated in vacuo, and the crude product was crystallized from diethyl ether. The obtained compounds in the form of hydrobromides were dissolved in water and neutralized with 2M NaOH to pH 7–8. The product was extracted with chloroform (4 × 15 mL), the solvent was evaporated under reduced pressure, and crystallized in diethyl ether.

2-(Cyclohexylamino)-5-methylthiazol-4(5*H*)-one (**3a**)

White powder; yield: 54.79%; m.p. 247–250 °C; ^1^H NMR (700 MHz, CDCl_3_): δ 12.45 (s, 1H), 4.29 (4, *J* = 7.7 Hz, 1H), 3.43 (s, 1H), 2.05–1.98 (m, 2H), 1.96–1.88 (m, 2H), 1.81 (d, *J* = 7.7 Hz, 3H), 1.77–1.69 (m, 2H), 1.68–1.62 (m, 2H), 1.46–1.36 (m, 3H). ^13^C NMR (100 Hz, CDCl_3_): δ 171.76, 171.45, 57.96, 44.72, 31.37, 24.54, 23.74, 17.99; HRMS: calc. for C_10_H_16_N*2*OS [M^+^ + 1]: 213.1062, found: 213.1061.

2-(Cyclohexylamino)-5-ethylthiazol-4(5*H*)-one (**3b**)

White powder; yield: 80.06%; m.p. 225–227 °C; ^1^H NMR: (700 MHz, CDCl_3_) δ 12.24 (s, 1H), 4.30 (dd, *J*_1_ = 4.2 Hz, *J*_2_ = 7.7 Hz, 1H), 3.47 (s, 1H), 2.30–2.23 (m, 1H), 2.14–2.06 (m, 1H), 2.05–1.98 (m, 2H), 1.96–1.88 (m, 2H), 1.77–1.70 (m, 2H), 1.67–1.59 (m, 2H), 1.44–1.38 (m, 3H), 1.15 (t, *J* = 7 Hz, 2H); ^13^C NMR (100 Hz, CDCl_3_): δ 171.55, 171.04, 57.99, 52.09, 31.38, 25.60, 24.50, 23.79, 10.83; HRMS: calc. for C_11_H_18_N_2_OS [M^+^ + 1]: 227.1218, found: 227.1224.

2-(Cyclohexylamino)-5-propylthiazol-4(5*H*)-one (**3c**)

White powder; yield: 82.75%; m.p. 228–230 °C; ^1^H NMR: (700 MHz, CDCl_3_) δ 12.17 (s, 1H), 4.32 (m, 1H), 3.46 (s, 1H), 2.28–2.18 (m, 1H), 2.07–1.99 (m, 2H), 1.99–1.95 (m, 1H), 1.94–1.87 (m, 2H), 1.78–1.69 (m, 2H), 1.68–1.58 (m, 2H), 1.58–1.47 (m, 2H), 1.45–1.33 (m, 3H), 1.04 (t, *J* = 7 Hz, 3H); ^13^C NMR (100 Hz, CDCl_3_): δ 171.92, 170.98, 59.77, 52.23, 32.54, 32.50, 25.57, 23.71, 10.80; HRMS: calc. for C_12_H_20_N_2_OS [M^+^ + 1]: 241.1375, found: 241.1381.

#### 3.2.2. Method B

First, a sodium methoxide solution was prepared by dissolving 0.092 g of sodium in 8 mL of anhydrous methanol. Then, 0.002 mol of *N*-cyclohexylthiourea (**1**) and 0.0022 mol of the appropriate 2-bromo ester (**2d**–**2e**) were added to the prepared solution. The reaction mixture was heated under reflux for 7 days (**3d**) and 14 days (**3e**). The course and completion of the reaction were monitored by TLC (chloroform/ethanol, 9:1). After the reaction was completed, the solvent was evaporated under reduced pressure, and the obtained mixture was dissolved in 15 mL of water and neutralized with 2M hydrochloric acid. The product was extracted with chloroform (4 × 20 mL), the solvent was evaporated in vacuo and the product was purified by column chromatography (chloroform/ethanol, 9:1) to give the desired pure product. The pure product obtained was recrystallized from diethyl ether.

2-(Cyclohexylamino)-5-isopropylthiazol-4(5*H*)-one (**3d**)

White powder; yield: 14.55%; m.p. 160–162 °C; ^1^H NMR (700 MHz, CDCl_3_): δ 4.21 (d, *J* = 3.5 Hz, 1H), 3.33–3.24 (m, 1H), 2.65–2.54 (m, 1H), 2.00–1.92 (m, 2H), 1.91–1.81 (m, 2H), 1.79–1.71 (m. 2H), 1.70–1.58 (m, 2H), 1.35–1.29 (m, 3H), 1.08 (d, *J* = 7.7, 3H), 0.91 (d, *J* = 6.3 Hz, 3H); ^13^C NMR (100 Hz, CDCl_3_): δ 187.09, 180.47, 62.89, 56.40, 32.45, 32.38, 30.50, 25.19, 24.82, 22.15. 16.30; HRMS: calc. for C_12_H_20_N_2_OS [M^+^ + 1]: 241.1375, found: 241.1376.

2-(Cyclohexylamino)-5,5-dimethylthiazol-4(5*H*)-one (**3e**)

White powder; yield: 11,78%; m.p. 218.5–221.3 °C; ^1^H NMR (700 MHz, CDCl3): δ 3.22–3.13 (m, 1H), 2.00–1.92 (m, 2H), 1.90–1.82 (m, 2H), 1.81–1.71 (m, 2H), 1.68–1.62 (m, 8H), 1.36–1.26 (m, 3H); ^13^C NMR (100 Hz, CDCl3): δ 191.05, 178.70, 59.79, 56.41, 32.42, 27.93, 25.24, 24.82; HRMS: calc. for C_11_H_19_N_2_OS [M^+^ + 1]: 227.1218, found: 227.1217.

#### 3.2.3. Method C

In a 108 mL reaction vessel were placed 0.002 mol *N*-cyclohexylthiourea (**1**), 0.0022 mol of the appropriate 2-bromoester (**2f**–**2g**), 0.374 mL of *N*-ethyldiisopropylamine, and 2 mL of anhydrous ethanol (in the case of the synthesis of compounds **3h**–**3i**, proportionally 4 times the amount of reagents was used). The reaction vessel was first heated in a microwave reactor for 15 min at 150–155 °C and then for 1 h at 155–160 °C. After cooling the reaction mixture, the solvent was evaporated, the residue was dissolved in 20 mL of water, and the pH was measured. The product was extracted with chloroform (4 × 20 mL). The organic fraction was dried over MgSO_4_, filtered, and the solvent was evaporated. Column chromatography purified the product (eluted with chloroform/ethanol 9:1).

2-(Cyclohexylamino)-5-phenylthiazol-4(5*H*)-one (**3f**)

White powder; yield: 74.68%; m.p. 208–210 °C; ^1^H NMR (700 MHz, CDCl_3_): δ 7.47–7.34 (m, 5H), 5.31 (dd, *J*_1_ = 7.0 Hz, *J*_2_ = 11.9 Hz, 1H), 3.39 (s, 1H), 2.20–1.97 (m, 2H), 1.96–1.82 (m, 2H), 1.82–1.70 (m, 2H), 1.68–1.61 (m, 1H), 1.46–1.30 (m, 4H); ^13^C NMR (100 Hz, CDCl_3_): δ 177.29, 175.44, 133.52, 129.34, 128.90, 128.42, 57.02, 55.90, 32.03, 24.65, 24.32; HRMS: calc. for C_15_H_18_N_2_OS [M^+^ + 1]: 275.1218, found: 275.1234.

5-(4-Bromophenyl)-2-(cyclohexylamino)thiazol-4(5*H*)-one (**3g**)

White powder; yield: 28.30%; m.p. 255 °C (dec.); ^1^H NMR (700 MHz, CDCl_3_): δ 7.57–7.47 (m, 2H), 7.27–7.22 (m, 2H), 5.23 (s, 1H), 3.41–3.32 (m, 1H), 2.10–1.97 (m, 2H), 1.96–1.84 (m, 2H), 1.84–1.68 (m, 2H), 1.68–1.60 (m, 1H), 1.43–1.22 (m, 4H); ^13^C NMR (100 Hz, CDCl_3_): δ 186.93, 177.69, 137.52, 132.05, 130.89, 121.36, 57.62, 54.68, 32.25, 25.36, 24,68; HRMS: calc. for C_15_H_18_N_2_OSBr [M^+^ + 1]: 353.0323, found:353.0323.

2-(Cyclohexylamino)-1-thia-3-azaspiro [4.5]dec-2-en-4-one (**3h**)

White powder; yield: 1.40%; m.p. 244.2–245.4 °C; ^1^H NMR (700 MHz, CDCl_3_): δ 3.28–3.20 (m, 1H), 2.13–2.04 (m, 2H), 1.99–1.90 (m, 4H), 1.88–1.71 (m, 8H), 1.68–1.62 (m, 1H), 1.46–1.22 (m 7H); ^13^C NMR (100 Hz, CDCl_3_): δ 190.71, 179.68, 68.90, 56.54, 36.65, 32.44, 25.14, 24.94, 24.85, 24.65; HRMS: calc. for C_12_H_20_N_2_OS [M^+^ + 1]: 267.1531, found: 267.1529.

6-(Cyclohexylamino)-5-thia-7-azaspiro [3.4]oct-6-en-8-one (**3i**)

White powder; yield: 34,62%; m.p. 196.8–197.3 °C; ^1^H NMR (700 MHz, CDCl_3_): δ 10.53 (br. s, 1H), 3.22–3.10 (m, 1H), 2.89–2.76 (m, 2H), 2.57–5.44 (m, 2H), 2.37–2.22 (m, 1H), 2.12–2.00 (m, 1H), 1.98–1.70 (m, 6H), 1.89–1.56 (m, 1H), 1.45–1.09 (m, 3H); ^13^C NMR (100 Hz, CDCl_3_): δ 190.74, 178.80, 60.65, 56.47, 34.20, 32.48, 25.28, 24.84, 16.92; HRMS: calc. for C_12_H_19_N_2_OS [M^+^ + 1]: 239.1218, found: 239.1217.

### 3.3. 11β-HSD Inhibition Assays

11β-Hydroxysteroid dehydrogenase type 1 and type 2 inhibition assays were performed as previously described [20]. For more details, see the Supplementary Materials.

### 3.4. Molecular Docking

All ligands (see Table 1) were prepared using Avogadro 1.1.1 [31] and optimized with the UFF force field [32] (5000 steps, conjugate gradient algorithm). Docking simulations were conducted using AutoDock Vina 1.2.2 software [33]. More details are given in our previous work [20].

### 3.5. Cell Culture

Human fibroblast (BJ; Cat# CRL-2522), human pancreatic cancer (PANC-1; Cat# CRL-1469), human breast cancer (MDA-MB-231; Cat# HTB-26); human brain glioma cells (U-118 MG; Cat# HTB-15), human melanoma (SK-MEL-30; Cat# ACC 151) and human colon carcinoma (Caco-2; Cat# HTB-37) were purchased from ATCC (Manassas, VA, USA) or DSMZ (Braunschweig, Germany). The cell lines were routinely maintained in the medium recommended by the manufacturer—MEM, DMEM, or RPMI (Corning, New York, NY, USA) with L-glutamine, 4.5 g/L glucose, and sodium pyruvate and supplemented with 10% FBS for BJ, PANC-1, MDA-MB-231, U-118 MG, and SK-MEL-30 or 20% FBS for Caco-2. All cell lines were cultured in a humidified CO_2_ incubator at 37 °C. For assays, cells were seeded at a constant density of 5 × 10^3^ cells/0.32 cm^2^ for Caco-2, PANC-1, MDA-MB-231, U-118 MG, and SK-MEL-30 or 2 × 10^3^ cells/0.32 cm^2^ for BJ.

### 3.6. MTS Assay

MTS assay was performed as described elsewhere [20]. Cell lines were seeded in a 96-well plate 24 h prior to treatment. Compounds **3a**–**3i** were tested in a 10–500 µM concentration range and treated for 72 h. Next, the MTS solution was added for **3h** and incubated at 37 °C. Absorbance was measured at 590 nm and 620 nm wavelengths using a Synergy H1 microplate reader (BioTek Instruments, San Diego, CA, USA).

### 3.7. Analysis of Intracellular Redox Homeostasis

Based on the MTS assay, a toxic concentration of 500 µM of each compound was selected for redox analysis for all cell lines. Cell lines were seeded at the standard density in 96-well black plates. Measurement of superoxide, nitric oxide, and free thiol levels was carried out using fluorogenic probes: dihydroethidium (Cayman Chemical, Ann Arbor, MI, USA, #12013), DAF-2 diacetate (Cayman Chemical, Ann Arbor, MI, USA, #85165), and Thiol Tracker Violet (Thermo Scientific, Waltham, MA, USA, #T10095). Next, cells were treated with the compounds at a concentration of 500 µM for 72 h. After incubation, probes were mixed and added to the cells at a final concentration of 5 µM each for 15 min at 37 °C. The signal measurement was taken with a Synergy H1 microplate reader (BioTek Instruments, CA, USA).

## 4. Conclusions

In the presented study, nine new derivatives of 2-(cyclohexylamino)thiazol-4-(5*H*)-one were successfully synthesized, and their biological activity was assessed. Compounds **3a**–**3i** were tested for their inhibitory activity and selectivity towards both isoforms of 11β-HSD (11β-HSD1 and 11β-HSD2). Out of nine compounds, as many as five compounds showed high inhibitory activity towards 11β-HSD1 (at a concentration of 10 µM, % inhibition of 11β-HSD1 was in the range of 85.72–93.99%). Derivative 3h showed the highest inhibitory activity towards 11β-HSD1 (IC_50_ = 0.04 µM). Moreover, this compound was characterized by better activity and selectivity than carbenoxolone used in the control. The results suggest that derivative 3h is an excellent candidate for further studies to elucidate its therapeutic potential fully. Furthermore, molecular docking analysis showed that the introduction of a large cyclohexyl substituent led to a favorable improvement in binding energy and increased ligand-protein affinity.

In the case of antitumor activity studies, the results indicate that most of the tested compounds do not inhibit the viability of pancreatic cancer (PANC-1) or glioma (U-118) cells and show only limited activity against breast cancer (MDA-MB-231), melanoma (SK-MEL-30), and human colon adenoma (Caco-2) cells. Interestingly, activity against MDA-MB-231, SK-MEL-30, and Caco-2 cell lines is observed only at relatively high concentrations of 200 µM. An exception is compound **3g**, which shows vigorous inhibitory activity against melanoma, breast cancer, and human colon adenoma cells. A decrease in Caco-2 cell viability was observed for compound **3g** at a concentration of 1 µM, while the viability of MDA-MB-231 cells was reduced at a concentration as low as 75 µM. Therefore, compound **3g** has the potential to become a lead compound in the design of new derivatives with anticancer properties, especially in the context of breast and colon cancer.

In summary, the above research results provide valuable information on the anticancer activity and 11β-HSD1 inhibition of 2-aminothiazol-4(5*H*)-one derivatives, highlighting their potential as multifunctional therapeutic agents.

## Figures and Tables

**Table 1:** The yields and melting points of pseudothiohydantoin derivatives **3a**–**3i**.

Compound	R_1_	R_2_	Synthesis Method	Isolated Yield (%)	Melting Point (°C)
**3a**	H	CH_3_	A	54.79 ^1^	164.2–165.3
**3b**	H	C_2_H_5_	A	80.06 ^1^	152.1–153.0
**3c**	H	C_3_H_7_	A	82.75 ^1^	147.5–146.8
**3d**	H	CH(CH_3_)_2_	B	14.55	160–162
**3e**	CH_3_	CH_3_	B	11.78	218.5–221.3
**3f**	H	C_6_H_5_	C	74.68	208–210
**3g**	H	C_6_H_4_*p*-Br	C	28.30	255 (dec.)
**3h**	-(CH_2_)_5_-		C	1.40	244.2–245.4
**3i**	-(CH_2_)_3_-		C	34.62	196.8–197.3

^1^ yield for the hydrobromide.

**Table 2:** 11β-HSD inhibitory activity of compounds **3a**–**3i**.

Compound	% of 11β-HSD1 Inhibition	IC_50_ 11β-HSD1	% of 11β-HSD2 Inhibition
**3a**	27.48 ± 2.12	>10	42.03 ± 2.50
**3b**	68.47 ± 4.28	0.57 ± 0.06	38.34 ± 9.96
**3c**	85.72 ± 4.43	0.4 ± 0.005	47.10 ± 0.82
**3d**	90.38 ± 2.74	0.08 ± 0.015	47.01 ± 1.67
**3e**	82.56 ± 1.79	0.3 ± 0.05	38.59 ± 5.56
**3f**	83.84 ± 4.53	0.17 ± 0.035	39.96 ± 8.34
**3g**	90.15 ± 1.59	0.07 ± 0.008	47.20 ± 1.17
**3h**	93.99 ± 2.35	0.04 ± 0.004	45.65 ± 5.28
**3i**	87.35 ± 0.99	0.09 ± 0.016	50.97 ± 0.73
**Control**	90.42 ± 1.86	0.08 ± 0.006	55.22 ± 0.13 ^a^ 46.82 ± 3.75 ^b^

^a^ for carbenoxolone, ^b^ for 11β-glycyrrhetinic acid.

## Data Availability

The original contributions presented in this study are included in the article and supplementary material. Further inquiries can be directed to the corresponding author.

## Supplementary Materials

The following supporting information can be downloaded at: https://www.mdpi.com/article/10.3390/ijms26188972/s1.

## References

[1] Seckl J.R., Walker B.R. (2001). Minireview: 11beta-hydroxysteroid dehydrogenase type 1—A tissue-specific amplifier of glucocorticoid action. Endocrinology.

[2] Chapman K., Holmes M., Seckl J. (2013). 11β-hydroxysteroid dehydrogenases: Intracellular gate-keepers of tissue glucocorticoid action. Physiol. Rev..

[3] Kupczyk D., Studzińska R., Kołodziejska R., Baumgart S., Modrzejewska M., Woźniak A. (2022). 11β-Hydroxysteroid dehydrogenase type 1 as a potential treatment target in cardiovascular diseases. J. Clin. Med..

[4] Scott J.S., Goldberg F.W., Turnbull A.V. (2014). Medicinal chemistry of inhibitors of 11β-hydroxysteroid dehydrogenase type 1 (11β-HSD1). J. Med. Chem..

[5] Dekker M.J., Tiemeier H., Luijendijk H.J., Kuningas M., Hofman A., de Jong F.H., Stewart P.M., Koper J.W., Lamberts S.W. (2012). The effect of common genetic variation in 11β-hydroxysteroid dehydrogenase type 1 on hypothalamic-pituitary-adrenal axis activity and incident depression. J. Clin. Endocrinol. Metab..

[6] Slattery D.A., Uzunov D.P., Cryan J.F. (2016). 11-β hydroxysteroid type 1 knockout mice display an antidepressant-like phenotype in the forced swim test. Acta Neuropsychiatr..

[7] Li H., Hu S., Wu R., Zhou H., Zhang K., Li K., Lin W., Shi Q., Chen H., Lv S. (2023). 11β-Hydroxysteroid dehydrogenase type 1 facilitates osteoporosis by turning on osteoclastogenesis through hippo signaling. Int. J. Biol. Sci..

[8] Schwab D., Sturm C., Portron A., Fuerst-Recktenwald S., Hainzl S., Jordan P., Stewart W.C., Tepedino M.E., DuBiner H. (2017). Oral administration of the 11β-hydroxysteroid-dehydrogenase type 1 inhibitor RO5093151 to patients with glaucoma: An adaptive, randomised, placebo-controlled clinical study. BMJ Open Ophthalmol..

[9] Taves M.D., Otsuka S., Taylor M.A., Donahue K.M., Meyer T.J., Cam M.C., Ashwell J.D. (2023). Tumors produce glucocorticoids by metabolite recycling, not synthesis, and activate Tregs to promote growth. J. Clin. Investig..

[10] Swatler J., Ju Y.J., Anderson A.C., Lugli E. (2023). Tumors recycle glucocorticoids to drive Treg-mediated immunosuppression. J. Clin. Investig..

[11] Poinot H., Dupuychaffray E., Arnoux G., Alvarez M., Tachet J., Ezzar O., Moore J., Bejuy O., Olesti E., Visconti G. (2023). Activation of endogenous glucocorticoids by HSD11B1 inhibits the antitumor immune response in renal cancer. Oncoimmunology.

[12] Saito R., Miki Y., Abe T., Miyauchi E., Abe J., Nanamiya R., Inoue C., Sato I., Sasano H. (2020). 11β hydroxysteroid dehydrogenase 1: A new marker for predicting response to immune-checkpoint blockade therapy in non-small-cell lung carcinoma. Br. J. Cancer.

[13] Melo L.M.N., Herrera-Rios D., Hinze D., Löffek S., Oezel I., Turiello R., Klein J., Leonardelli S., Westedt I.V., Al-Matary Y. (2023). Glucocorticoid activation by HSD11B1 limits T cell-driven interferon signaling and response to PD-1 blockade in melanoma. J. Immunother. Cancer.

[14] Lee J.H., Bok J.H., Park S.B., Pagire H.S., Na Y.J., Rim E., Jung W.H., Song J.S., Kang N.S., Seo H.W. (2020). Optimization of cyclic sulfamide derivatives as 11β-hydroxysteroid dehydrogenase 1 inhibitors for the potential treatment of ischemic brain injury. Bioorg. Med. Chem. Lett..

[15] Joharapurkar A., Dhanesha N., Shah G., Kharul R., Jain M. (2012). 11β-hydroxysteroid dehydrogenase type 1: Potential therapeutic target for metabolic syndrome. Pharmacol. Rep..

[16] Leiva R., Grinan-Ferre C., Seira C., Valverde E., McBride A., Binnie M., Pérez B., Luque F.J., Pallàs M., Bidon-Chanal A. (2017). Design, synthesis and in vivo study of novel pyrrolidine-based 11β-HSD1 inhibitors for age-related cognitive dysfunction. Eur. J. Med. Chem..

[17] Zhang C., Xu M., He C., Zhuo J., Burns D.M., Qian D.Q., Lin Q., Li Y.L., Chen L., Shi E. (2022). Discovery of 1′-(1-phenylcyclopropane-carbonyl)-3H-spiro[isobenzofuran-1,3′-pyrrolidin]-3-one as a novel steroid mimetic scaffold for the potent and tissue-specific inhibition of 11β-HSD1 using a scaffold-hopping approach. Bioorg. Med. Chem. Lett..

[18] Shao L.D., Bao Y., Shen Y., Su J., Leng Y., Zhao Q.S. (2017). Synthesis of selective 11β-HSD1 inhibitors based on dammarane scaffold. Eur. J. Med. Chem..

[19] Gao Q., Kimura R.E., Zhang X., Nam J., Amore B.M., Hickman D., Greg Slatter J., Emery M.G. (2014). Intestinal and hepatic first-pass extraction of the 11β-HSD1 inhibitor AMG 221 in rats with chronic vascular catheters. Xenobiotica.

[20] Baumgart S., Kupczyk D., Archała A., Koszła O., Sołek P., Płaziński W., Płazińska A., Studzińska R. (2023). Synthesis of novel 2-(cyclopentylamino)thiazol-4(5*H*)-one derivatives with potential anticancer, antioxidant, and 11β-HSD inhibitory activities. Int. J. Mol. Sci..

[21] Studzińska R., Kupczyk D., Płaziński W., Baumgart S., Bilski R., Paprocka R., Kołodziejska R. (2021). Novel 2-(adamantan-1-yloamino)thiazol-4(5*H*)-one derivatives and their inhibitory activity towards 11β-HSD1—Synthesis molecular docking and in vitro studies. Int. J. Mol. Sci..

[22] Kupczyk D., Studzińska R., Baumgart S., Bilski R., Kosmalski T., Kołodziejska R., Woźniak A. (2021). A novel N-tert-butyl derivatives of pseudothiohydantoin as potential target in anti-cancer therapy. Molecules.

[23] Mądra-Gackowska K., Baumgart S., Jędrzejewski M., Studzińska R., Szeleszczuk Ł., Gackowski M. (2025). Computational QSAR study of novel 2-aminothiazol-4(5*H*)-one derivatives as 11β-HSD1 inhibitors. J. Comput. Aided. Mol. Des..

[24] Studzińska R., Kołodziejska R., Płaziński W., Kupczyk D., Kosmalski T., Jasieniecka K., Modzelewska-Banachiewicz B. (2019). Synthesis of the N-methyl derivatives of 2-aminothiazol-4(5*H*)-one and their interactions with 11βHSD1-molecular modeling and in vitro studies. Chem. Biodivers..

[25] Kupczyk D., Studzińska R., Bilski R., Baumgart S., Kołodziejska R., Woźniak A. (2020). Synthesis of novel 2-(isopropylamino)thia-zol-4(5*H*)-one derivatives and their inhibitory activity of 11β-HSD1 and 11β-HSD2 in aspect of carcinogenesis prevention. Molecules.

[26] Studzińska R., Kołodziejska R., Kupczyk D., Płaziński W., Kosmalski T. (2018). A novel derivatives of thiazol-4(5*H*)-one and their activity in the inhibition of 11β-hydroxysteroid dehydrogenase type 1. Bioorg. Chem..

[27] Wan Y., Long J., Gao H., Tang Z. (2010). 2-Aminothiazole: A privileged scaffold for the discovery of anti-cancer agents. Eur. J. Med. Chem..

[28] Baell J.B., Holloway G.A. (2010). New substructure filters for removal of pan assay interference compounds (PAINS) from screening libraries and for their exclusion in bioassays. J. Med. Chem..

[29] Rai M., Singh A.V., Paudel N., Kanase A., Falletta E., Kerkar P., Heyda J., Barghash R.F., Pratap Singh S., Soos M. (2023). Herbal concoction Unveiled: A computational analysis of phytochemicals’ pharmacokinetic and toxicological profiles using novel approach methodologies (NAMs). Curr. Res. Toxicol..

[30] SwissADME. http://swissadme.ch.

[31] Hanwell M.D., Curtis D.E., Lonie D.C., Vandermeersch T., Zurek E., Hutchison G.R.J. (2012). Avogadro: An Advanced Semantic Chemical Editor, Visualization, and Analysis Platform. J. Cheminform..

[32] Rappe A.K., Casewit C.J., Colwell K.S., Goddard W.A., Skiff W.M. (1992). UFF, a full periodic table force field for molecular mechanics and molecular dynamics simulations. J. Am. Chem. Soc..

[33] Trott O., Olson A.J. (2010). AutoDock Vina: Improving the speed and accuracy of docking with a new scoring function, efficient optimization, and multithreading. J. Comput. Chem..

