# Supplementary material for: New Derivatives of 2-(Cyclohexylamino)thiazol-4(5H)-one as Strong Inhibitors of 11β-Hydroxysteroid Dehydrogenase Type 1: Synthesis, Antiproliferative and Redox-Modulating Activity
Source: Int J Mol Sci. 2025 Sep 15;26(18):8972. doi: 10.3390/ijms26188972 (PMC12470129; doi:10.3390/ijms26188972)
Supplement: Supplementary file 1 [file ijms-26-08972-s001.zip › ijms-3831192-supplementary.pdf]

Supplementary Materials

(A). Detailed protocols of enzyme inhibition assays

1. Inhibition of 11β-HSD Assays

1.1. 11β-HSD1

Solvents and reagents: carbenoxolone (disodium salt) (Sigma-Aldrich, Japan), cortisone, NADPH tetrasodium salt, phosphate buffer powder, (Sigma-Aldrich, Poznań, Poland), Pooled human liver microsomes, mixed gender, 1 mL, 20 mg/mL Lot No.1410013 - XenoTech, Cortisol Elisa Ref DkO001 Lot No. 5671A (DiaMetra, Spello, Italy), ELISA Kit for 11-Beta-Hydroxysteroid Dehydrogenase Type 1 Lot No. L211008799 – (Cloud-Clone Corp., Wuhan, China), PBS Lot No. H161008 (Pan Biotech, Aidenbach, Germany).

To study the inhibitory effect of compounds **3a** – **3i** on the conversion of cortisone to cortisol, human liver microsomes were used as a source of the 11β-HSD1 enzyme. Standard 96-well microplates were filled with a reagent mixture: cortisone/NADPH (20 µl, to obtain a final concentration of 200 nM / 2 µM), microsomes (10 µl, 1.13 µg of 11β-HSD1 in 1 ml) in PBS solution (final amount 2.5 µg), phosphate buffer (60 µl, pH 7.4), and 10 µl of compounds **3a** – **3j** dissolved in a mixture containing 1% DMSO and 99% water. The resulting solution (final volume 100 µl) was incubated for 2.5 h at 37°C. To stop the reaction, 10 µl of a 100 µM solution of 18β-glycyrrhetinic acid in PBS was added. Cortisol levels resulting from the reaction were measured using a commercially available ELISA kit.

1.2. 11β-HSD2

Solvents and reagents: 18-beta-glycyrrhetinic acid – (Acros Organic, Geel, Belgium), cortisone, NAD cofactor, phosphate buffer powder (Sigma-Aldrich, Poznań, Poland), Human Kidney Microsomes, mixed gender, 0.5 mL, 10 mg/mL Lot No. 1710160 XenoTech, Cortisol Elisa Ref DkO001 Lot No. 5671A - (DiaMetra, Spello, Italy), Enzyme-Linked Immunosorbent Assay (ELISA) Kit for 11-Beta-Hydroxysteroid Dehydrogenase Type 2 Lot No. L191113457 - (Cloud-Clone Corp., Wuhan, China), PBS Lot No. H161008 (Pan Biotech, Aidenbach, Germany).

To study the inhibitory effect of compounds **3a** – **3i** on the conversion of cortisol to cortisone, human kidney microsomes were used as a source of the 11β-HSD2 enzyme. Standard 96-well microplates were filled with a reagent mixture: cortisol/NAD+ (20 µL, to obtain a final concentration of 200 nM / 2 µM), microsomes (10 µL, 0.127 µg of 11β-HSD2 in 1 ml) in PBS solution (final concentration 2.5 µg), phosphate buffer (60 µL, pH 7.4), and 10 µL of compounds **3a** – **3j** dissolved in a mixture containing 1% DMSO and 99% water. The resulting solution (final volume of 100 µL) was incubated for 2.5 h at 37°C. To stop the reaction, 10 µl of a 100 µM carbenoxolone solution in PBS was added. Unreacted cortisol levels were measured using a commercially available ELISA kit.

1.3. Determination of IC_{50}

Calibration curves for determining IC_{50} values for compounds **3a**-**3i** were prepared using solutions at concentrations of 0.078, 0.156, 0.3125, 0.625, 1.25, 2.5, 5.0, and 10.0 µM using the standard procedure and conditions described in the sections above. Analogous assays without added inhibitors were performed as controls. The IC_{50} value, defined as the concentration of inhibitor causing a 50% reduction in cortisol or cortisone, was read directly from the graph.

(B). Spectral data

S1. ¹H NMR spectra of compounds 3a – 3i

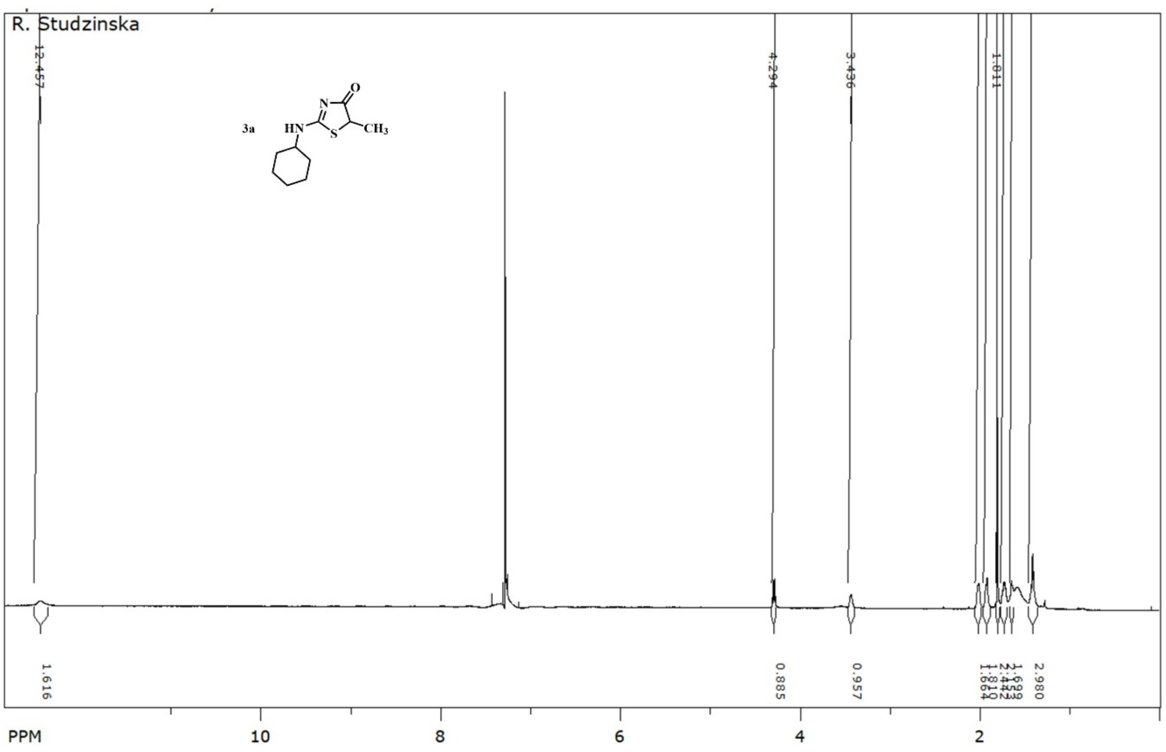

Figure S1. ¹H NMR spectra of compound **3a**

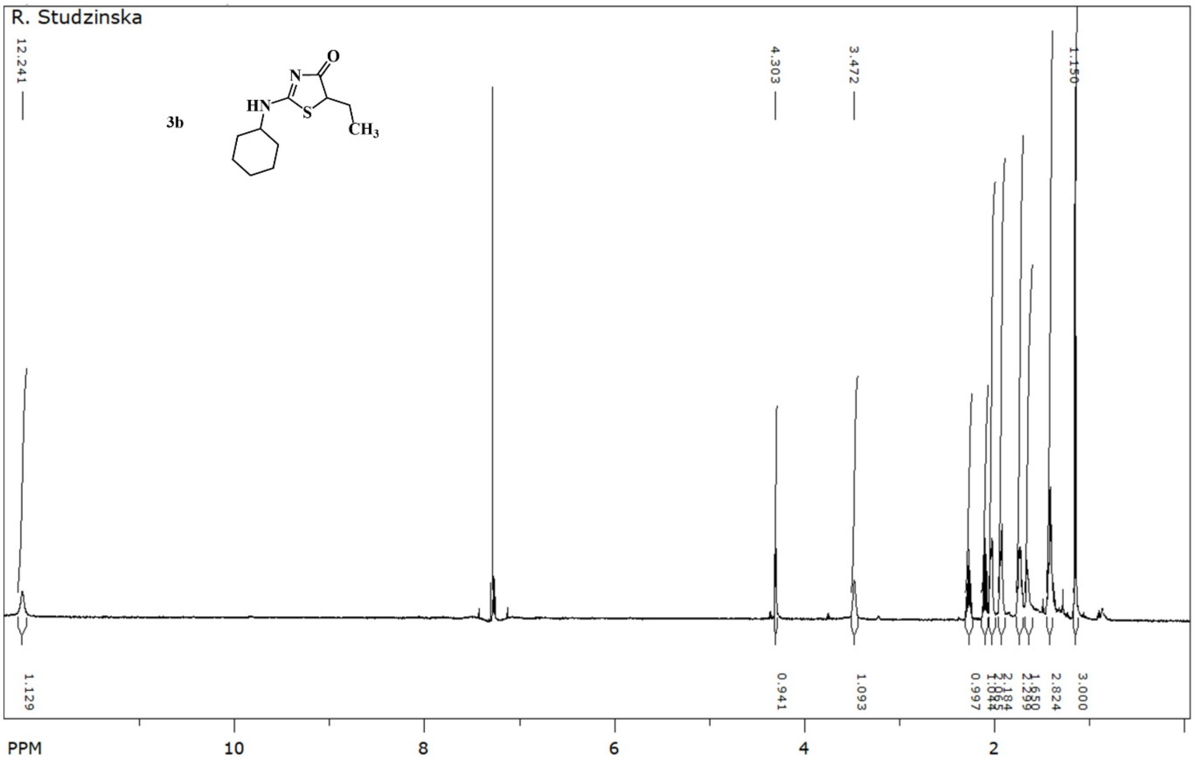

Figure S2. ^{1}H NMR spectra of compound **3b**

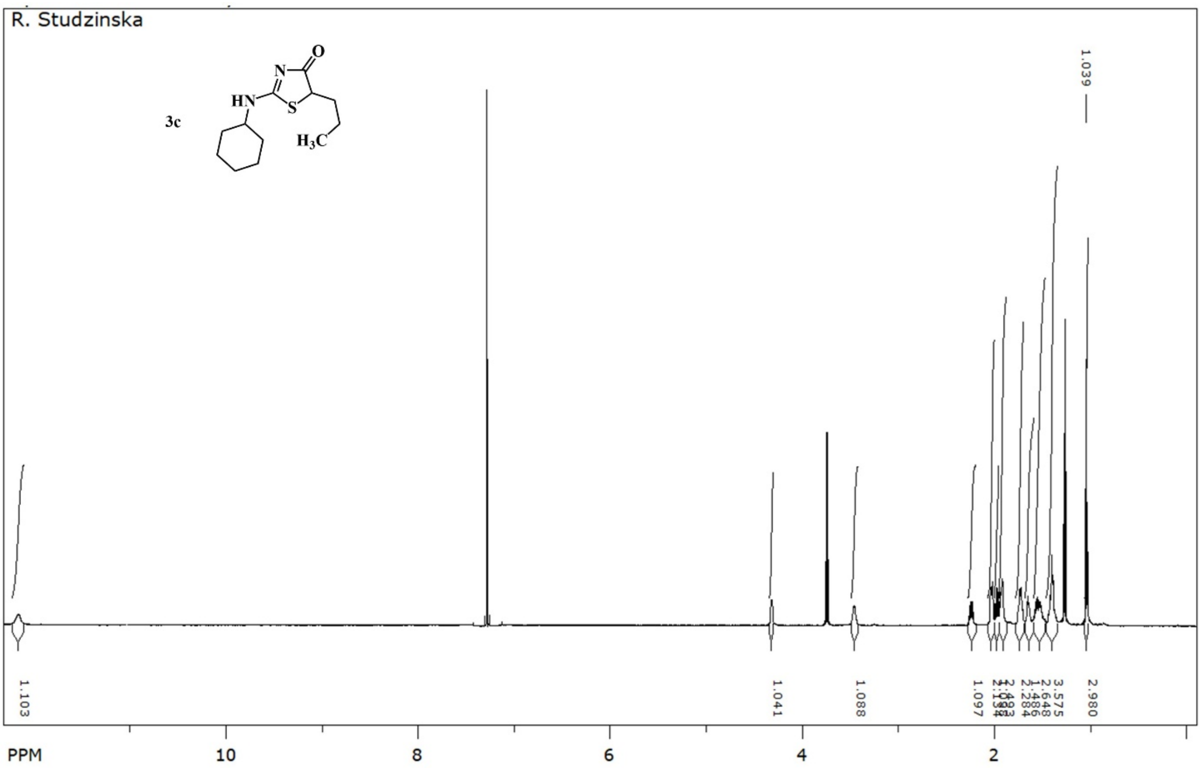

Figure S3. ^{1}H NMR spectra of compound **3c**

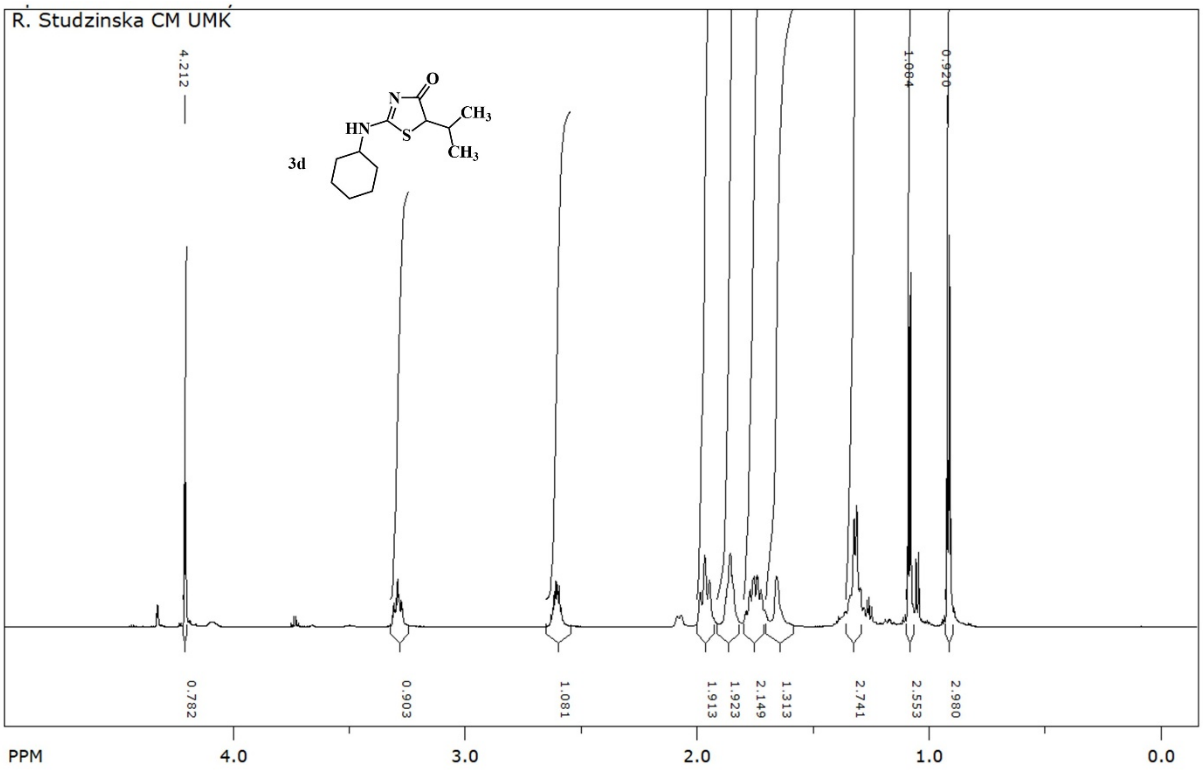

Figure S4. ^{1}H NMR spectra of compound **3d**

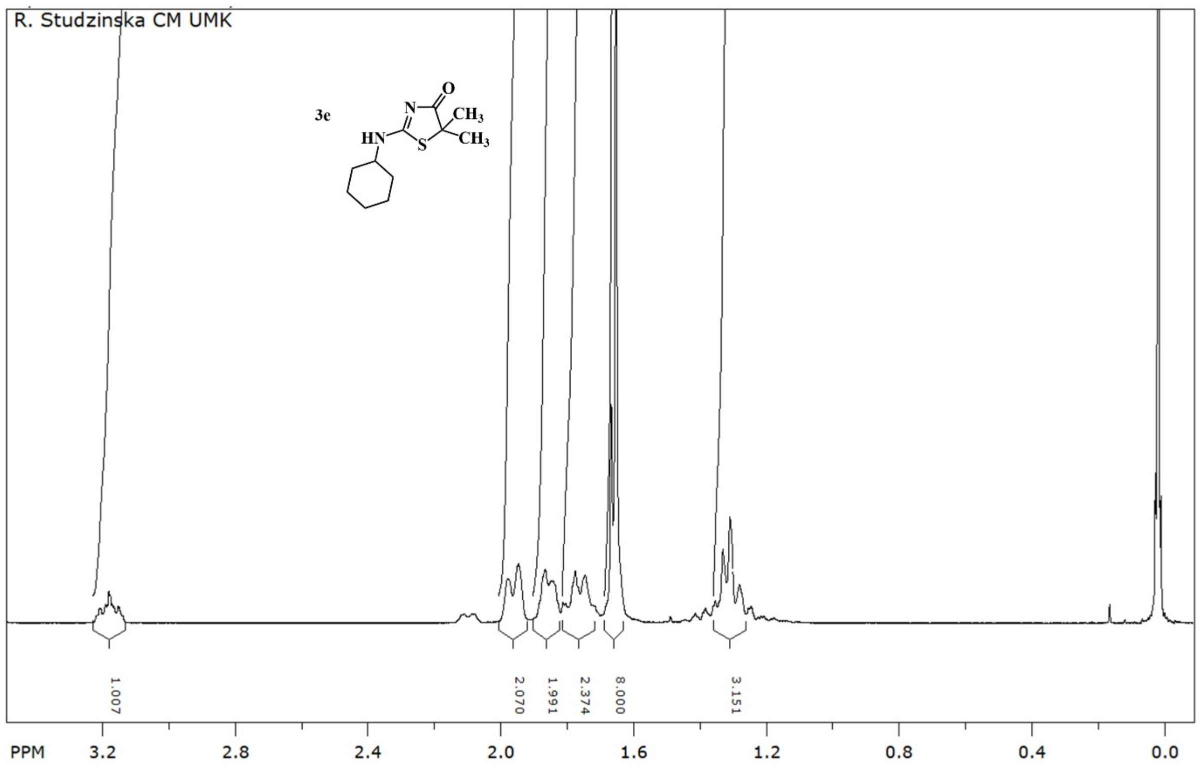

Figure S5. ^{1}H NMR spectra of compound **3e**

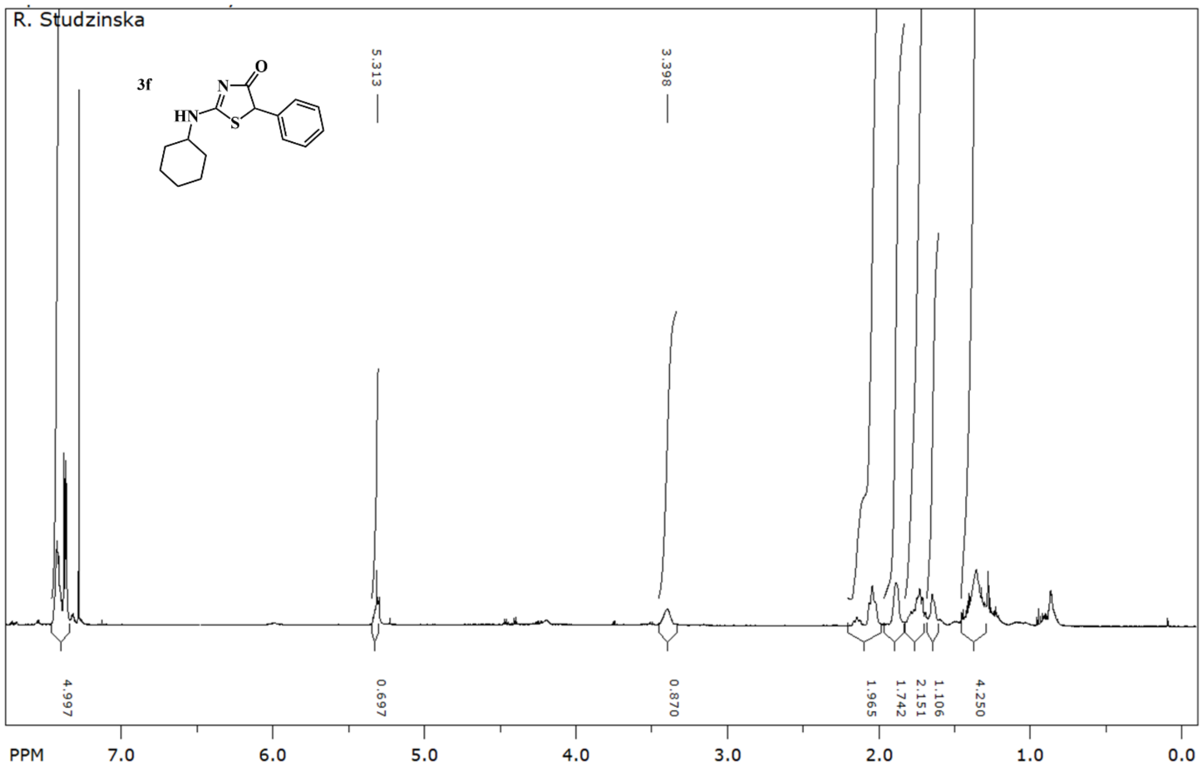

Figure S6. ^{1}H NMR spectra of compound **3f**

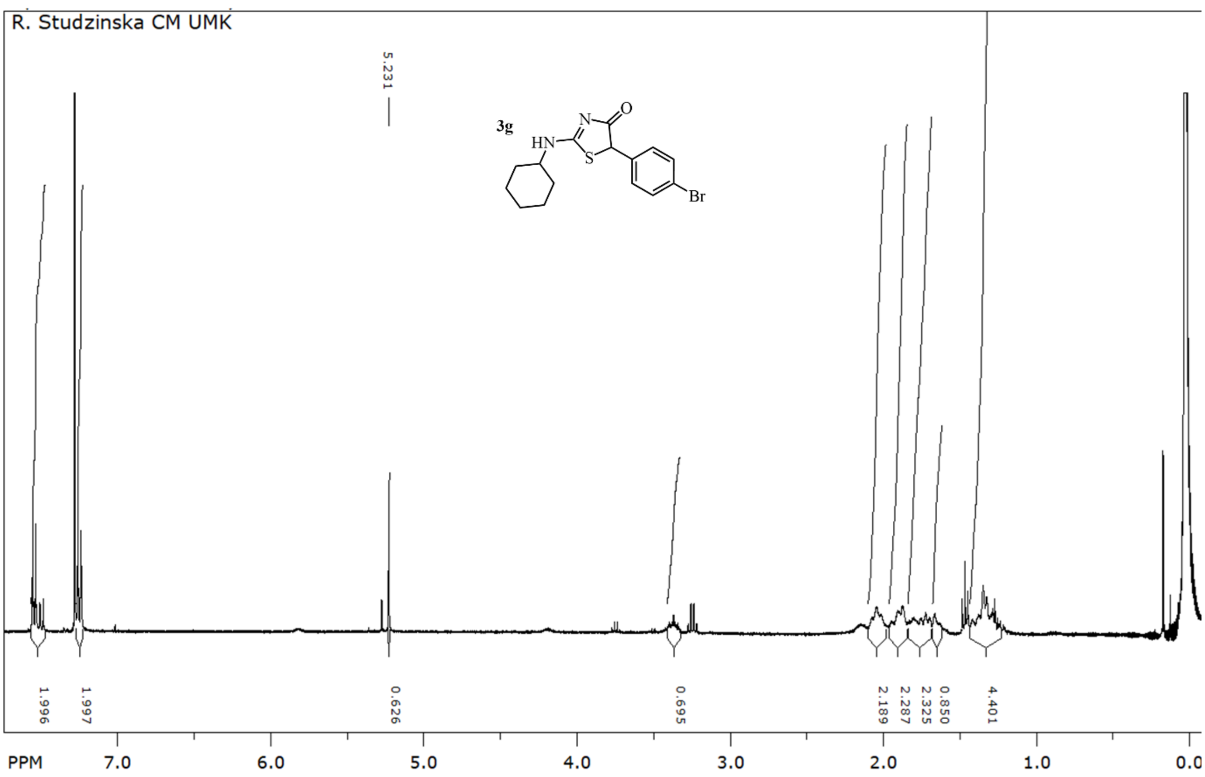

Figure S7. ^{1}H NMR spectra of compound **3g**

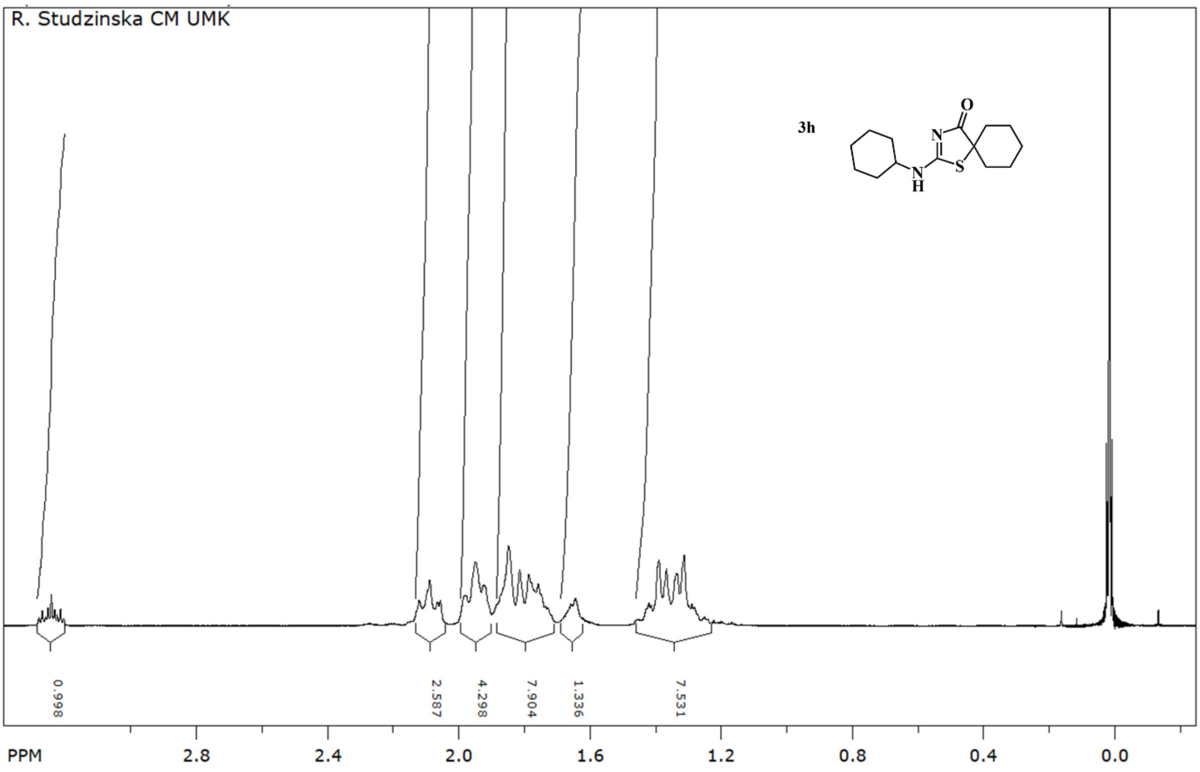

Figure S8. ^{1}H NMR spectra of compound **3h**

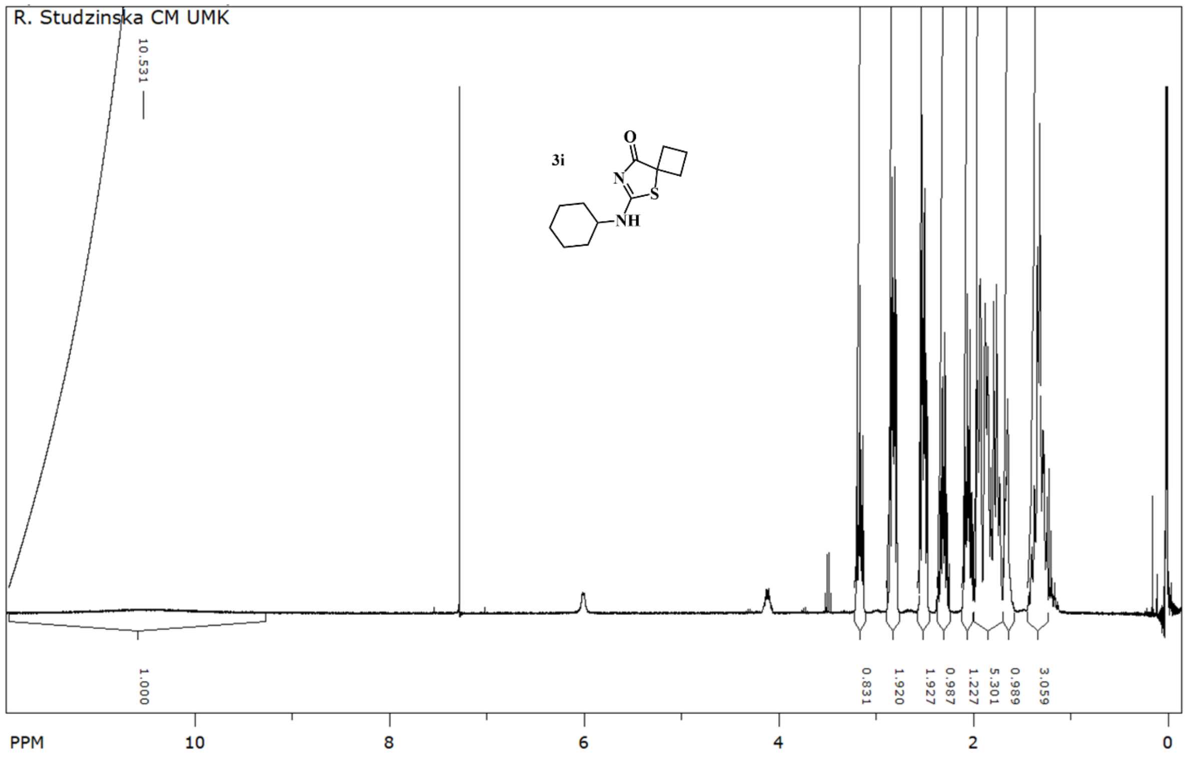

Figure S9. ^{1}H NMR spectra of compound **3i**

S2. 13C NMR spectra of compounds 3a – 3i

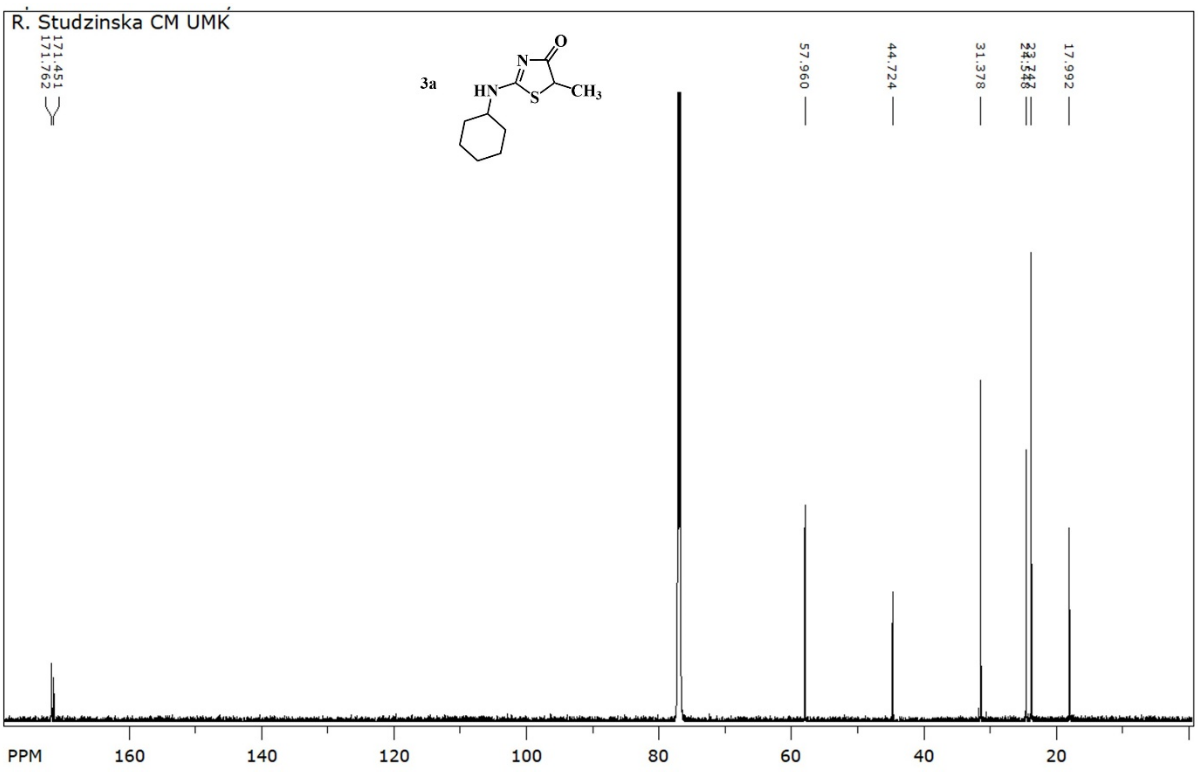

Figure S10. ^{13}C NMR spectra of compounds **3a**

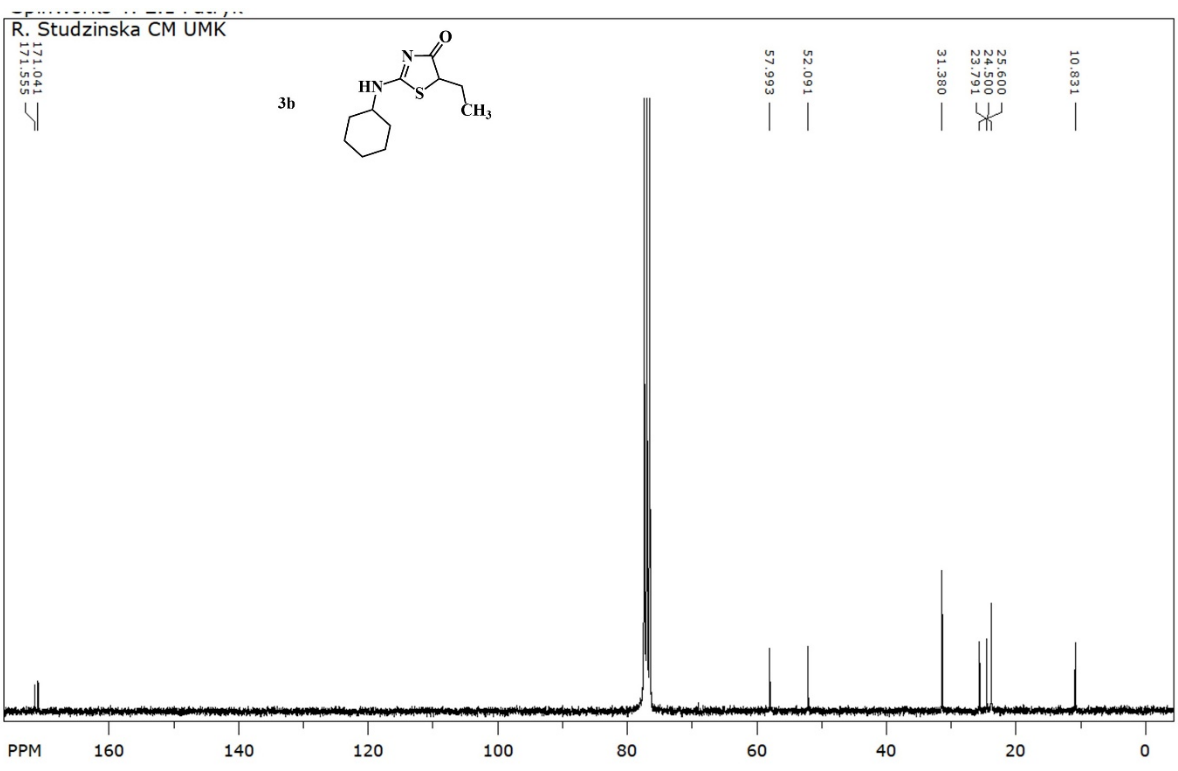

Figure S11. ^{13}C NMR spectra of compounds **3b**

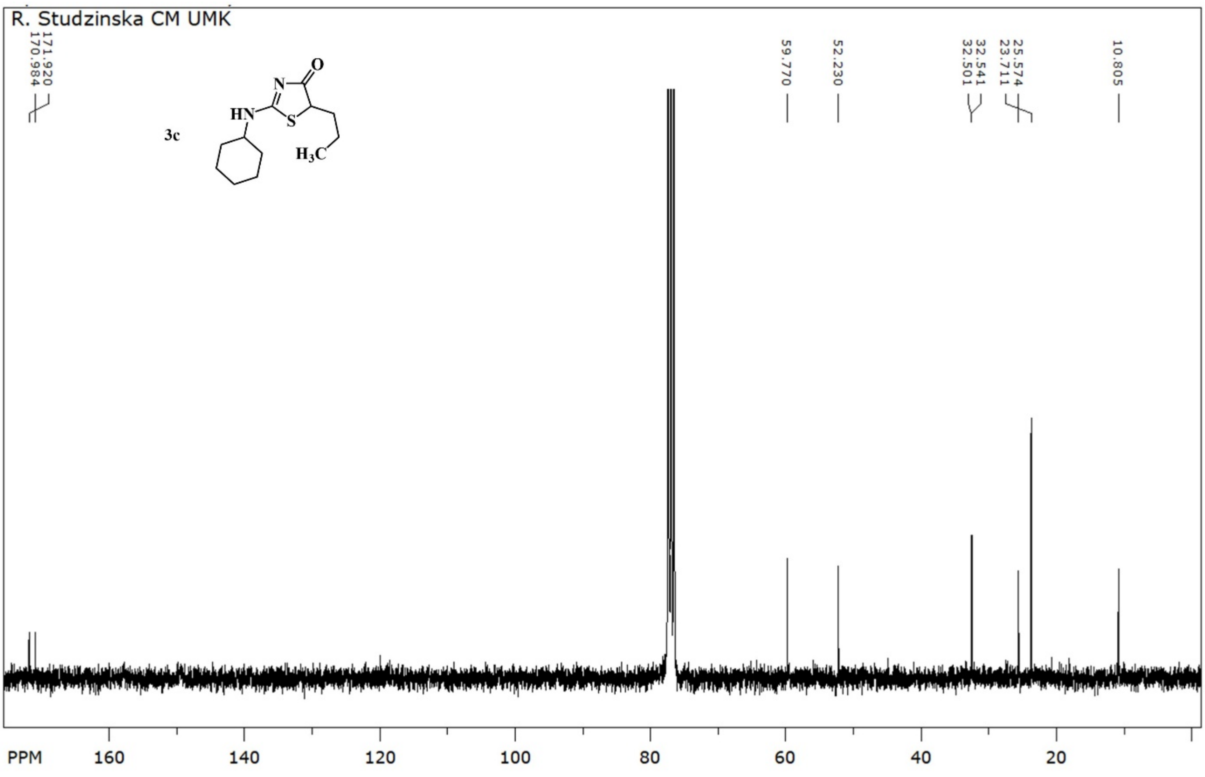

Figure S12. ^{13}C NMR spectra of compounds **3c**

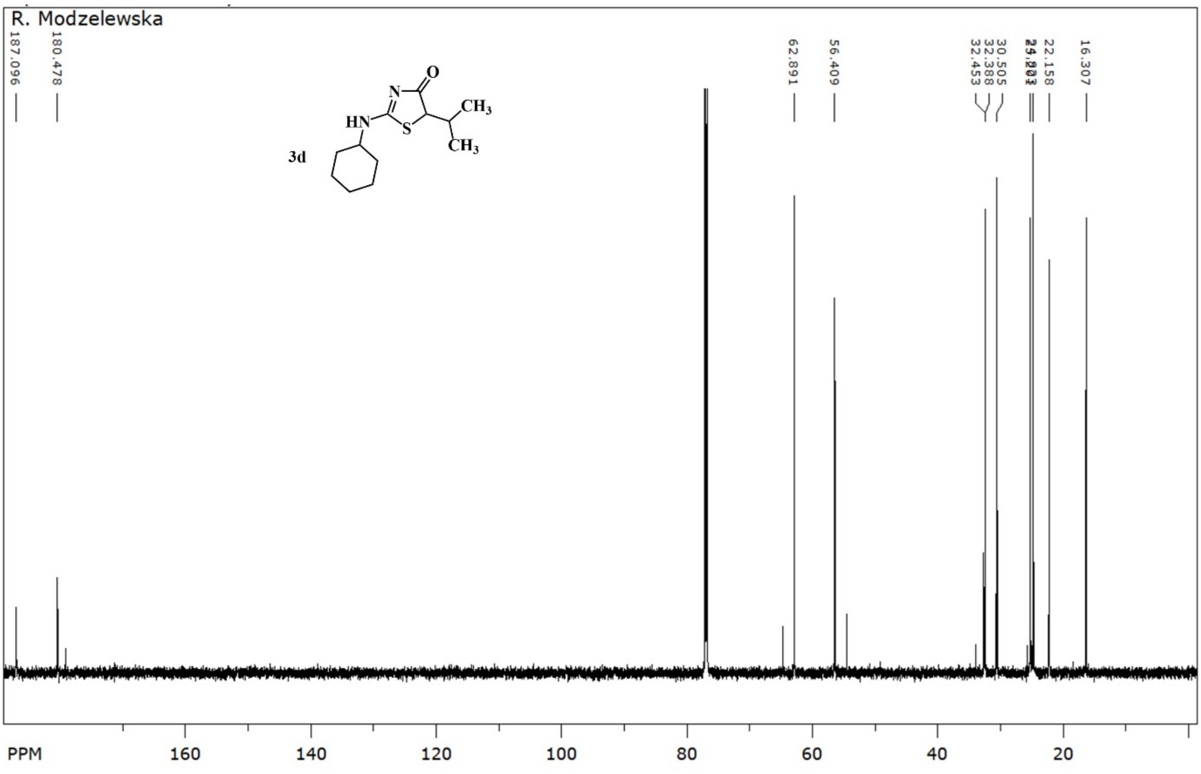

Figure S13. ^{13}C NMR spectra of compounds **3d**

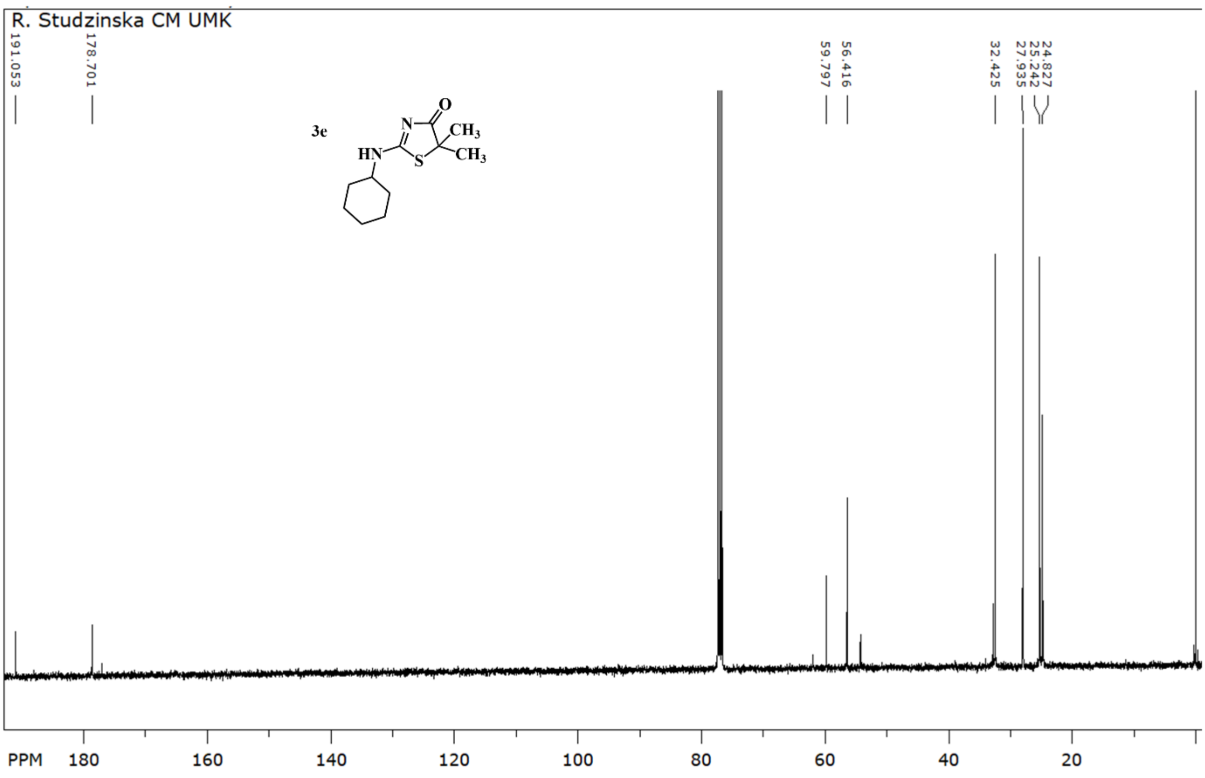

Figure S14. ^{13}C NMR spectra of compounds **3e**

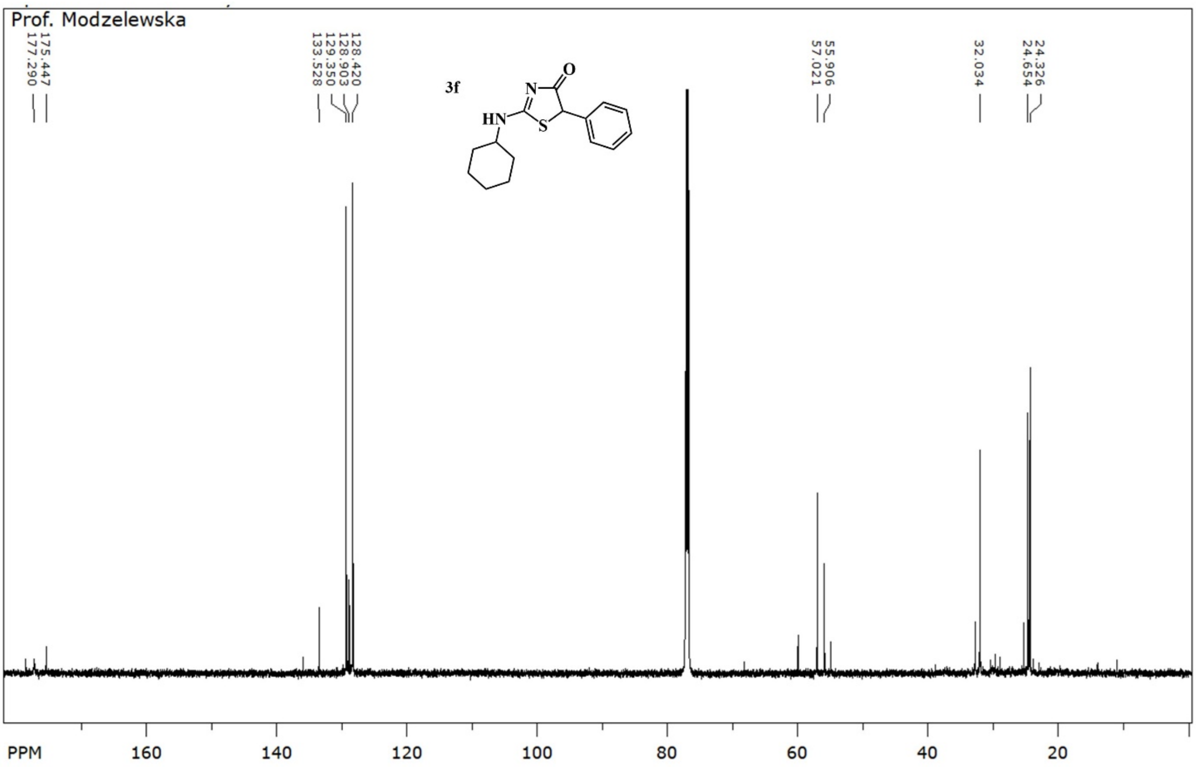

Figure S15. ^{13}C NMR spectra of compounds **3f**

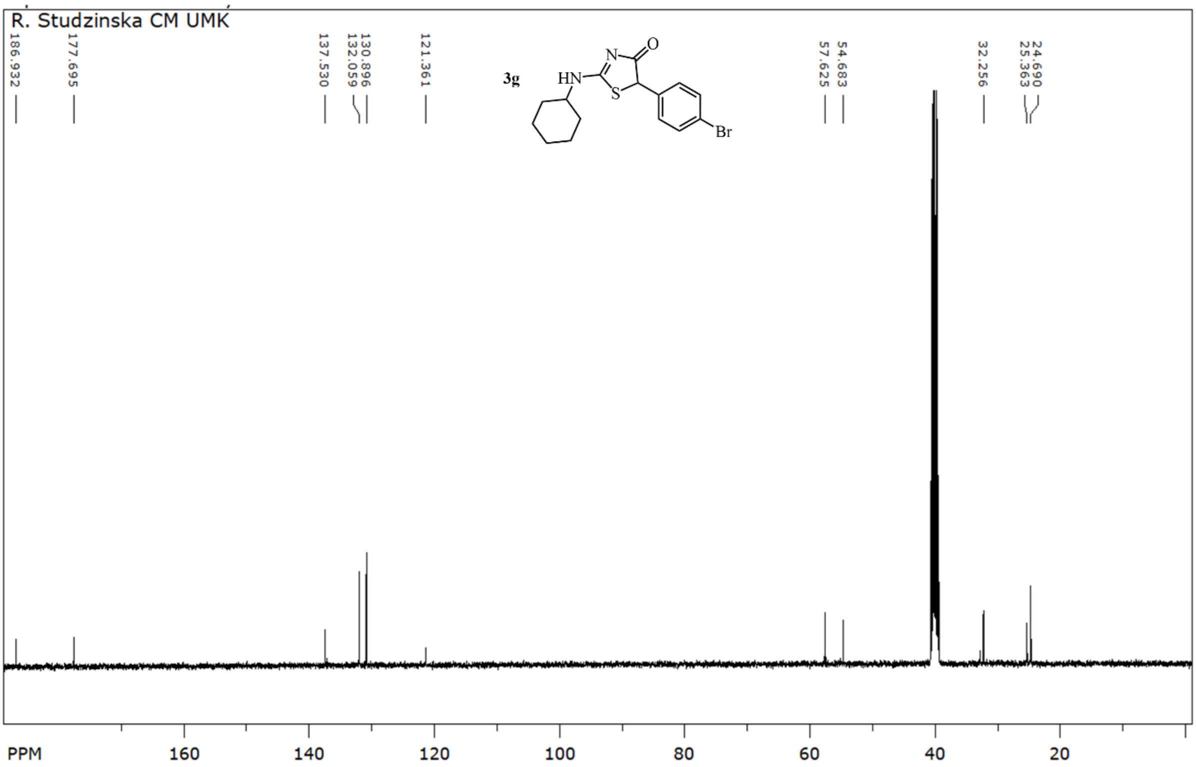

Figure S16. ^{13}C NMR spectra of compounds **3g**

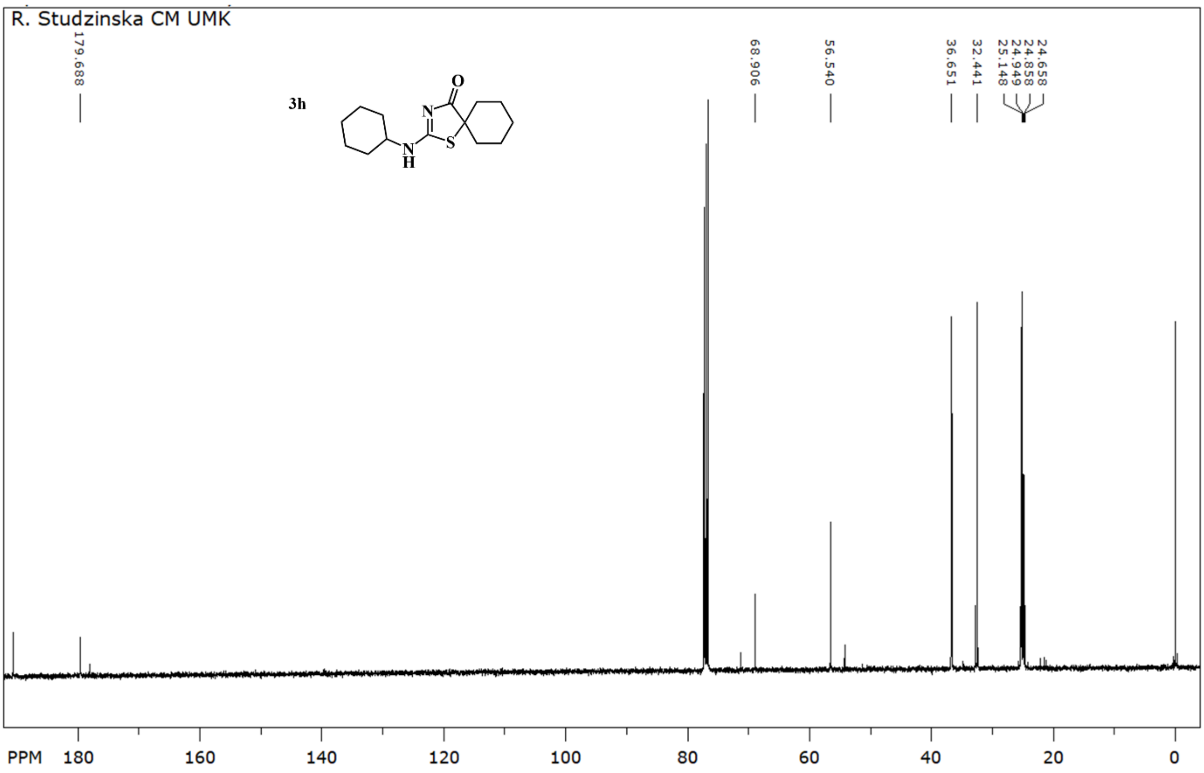

Figure S17. ^{13}C NMR spectra of compounds **3h**

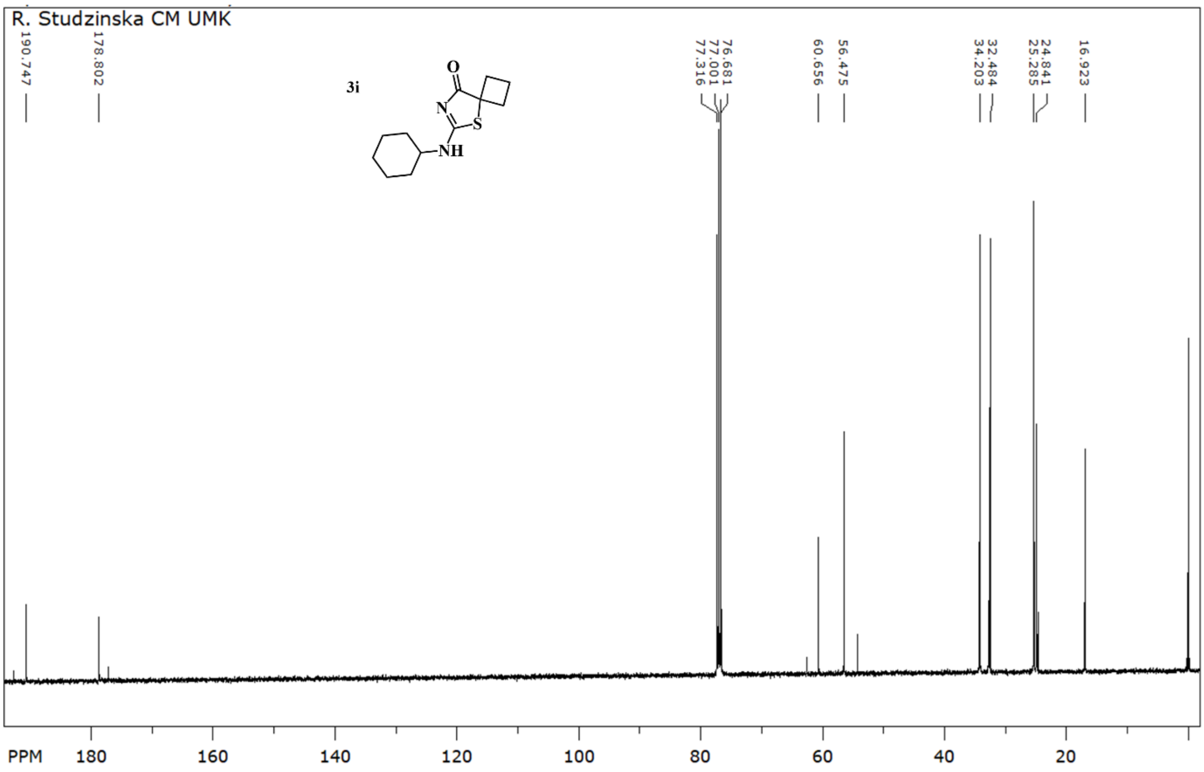

Figure S18. ^{13}C NMR spectra of compounds **3i**

S3. HRMS spectra of compounds 3a – 3i

Elemental Composition Report

Page 1

Single Mass Analysis

Tolerance = 10.0 PPM / DBE: min = -1.5, max = 70.0
Element prediction: Off
Number of isotope peaks used for i-FIT = 9

Monoisotopic Mass, Even Electron Ions
27 formula(e) evaluated with 1 results within limits (all results (up to 1000) for each mass)
Elements Used:
C: 0-20 H: 0-25 N: 0-2 O: 0-1 S: 0-1

3a

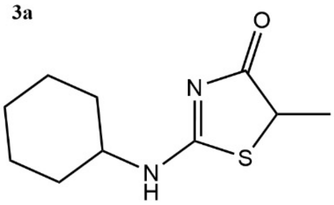

200526_Patryk_1A 37 (0.391) Cm (37:40-2:8)

TOF MS ES+
2.60e+006

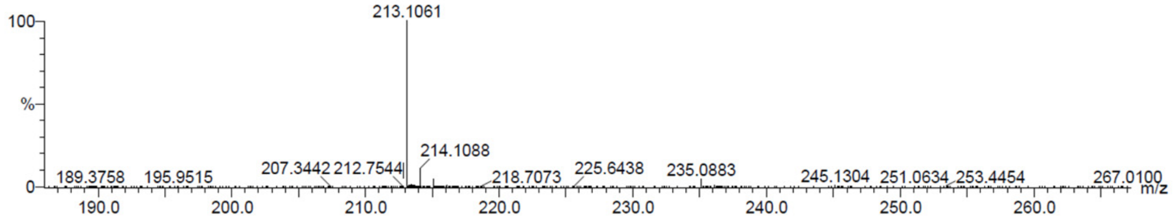

		mDa	PPM	DBE				
Minimum:				-1.5				
Maximum:		5.0	10.0	70.0				
**Mass**	**Calc. Mass**	**mDa**	**PPM**	**DBE**	**i-FIT**	**Norm**	**Conf(%)**	**Formula**
213.1061	213.1062	-0.1	-0.5	3.5	1153.8	n/a	n/a	C10 H17 N2 O S

Figure S19. HRMS spectra of compound **3a**

Elemental Composition Report

Page 1

Single Mass Analysis

Tolerance = 10.0 PPM / DBE: min = -1.5, max = 70.0
Element prediction: Off
Number of isotope peaks used for i-FIT = 9

Monoisotopic Mass, Even Electron Ions
28 formula(e) evaluated with 1 results within limits (all results (up to 1000) for each mass)
Elements Used:
C: 0-20 H: 0-25 N: 0-2 O: 0-1 S: 0-1

3b

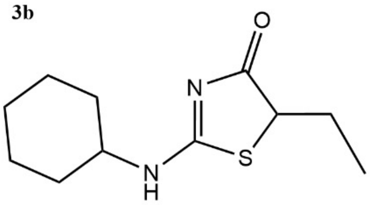

200526_Patryk_2A 12 (0.142) Cm (11:12-2:6)

TOF MS ES+
5.42e+006

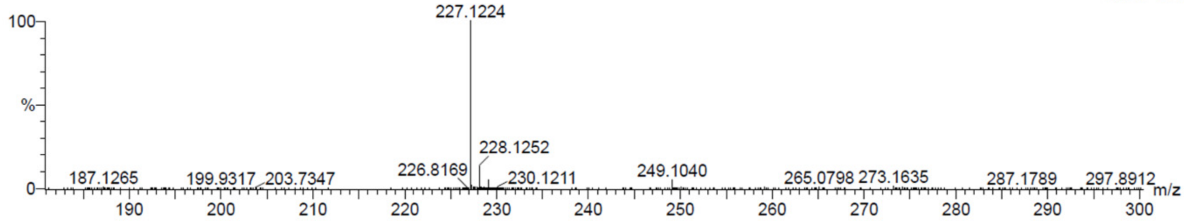

		mDa	PPM	DBE				
Minimum:				-1.5				
Maximum:		5.0	10.0	70.0				
**Mass**	**Calc. Mass**	**mDa**	**PPM**	**DBE**	**i-FIT**	**Norm**	**Conf(%)**	**Formula**
227.1224	227.1218	0.6	2.6	3.5	1076.3	n/a	n/a	C11 H19 N2 O S

Figure S20. HRMS spectra of compound **3b**

Elemental Composition Report

3c

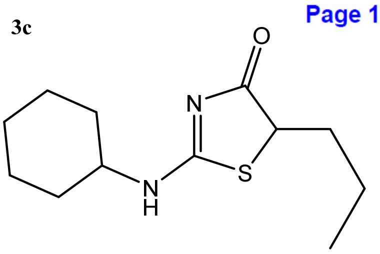

Page 1

Single Mass Analysis

Tolerance = 10.0 PPM / DBE: min = -1.5, max = 70.0
Element prediction: Off
Number of isotope peaks used for i-FIT = 9

Monoisotopic Mass, Even Electron Ions
29 formula(e) evaluated with 1 results within limits (all results (up to 1000) for each mass)
Elements Used:
C: 0-20 H: 0-25 N: 0-2 O: 0-1 S: 0-1

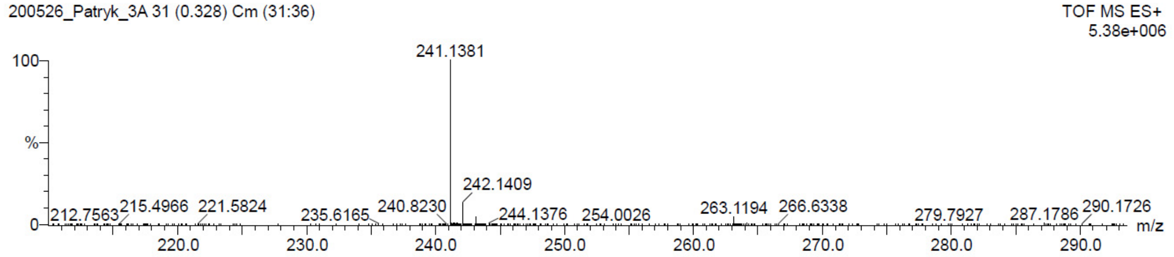

Minimum:				-1.5				
Maximum:		5.0	10.0	70.0				
Mass	Calc. Mass	mDa	PPM	DBE	i-FIT	Norm	Conf(%)	Formula
241.1381	241.1375	0.6	2.5	3.5	1170.7	n/a	n/a	C12 H21 N2 O S

Figure S21. HRMS spectra of compound **3c**

Elemental Composition Report

3d

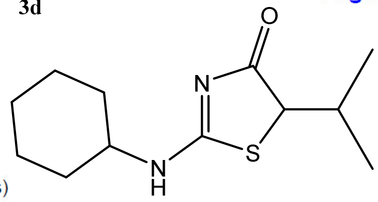

Page 1

Single Mass Analysis

Tolerance = 10.0 PPM / DBE: min = -1.5, max = 70.0
Element prediction: Off
Number of isotope peaks used for i-FIT = 9

Monoisotopic Mass, Even Electron Ions
29 formula(e) evaluated with 1 results within limits (all results (up to 1000) for each mass)
Elements Used:
C: 0-20 H: 0-25 N: 0-2 O: 0-1 S: 0-1

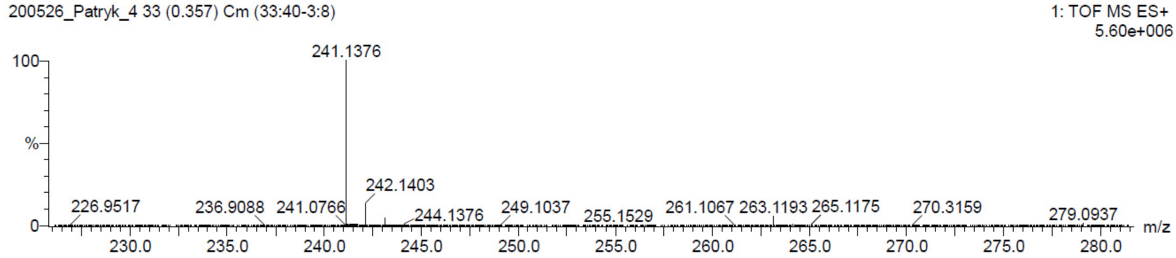

Minimum:				-1.5				
Maximum:		5.0	10.0	70.0				
Mass	Calc. Mass	mDa	PPM	DBE	i-FIT	Norm	Conf(%)	Formula
241.1376	241.1375	0.1	0.4	3.5	2694.7	n/a	n/a	C12 H21 N2 O S

Figure S22. HRMS spectra of compound **3d**

Elemental Composition Report

3e

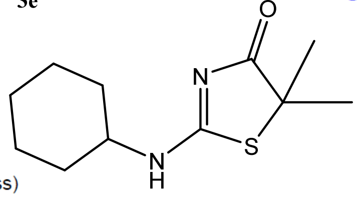

Page 1

Single Mass Analysis

Tolerance = 5.0 PPM / DBE: min = -1.5, max = 80.0
Element prediction: Off
Number of isotope peaks used for i-FIT = 9

Monoisotopic Mass, Even Electron Ions
207 formula(e) evaluated with 1 results within limits (all results (up to 1000) for each mass)
Elements Used:
C: 0-60 H: 0-70 N: 0-6 O: 0-4 S: 0-1
210318_5_1_PatrykA 21 (0.231) Cm (21:29)

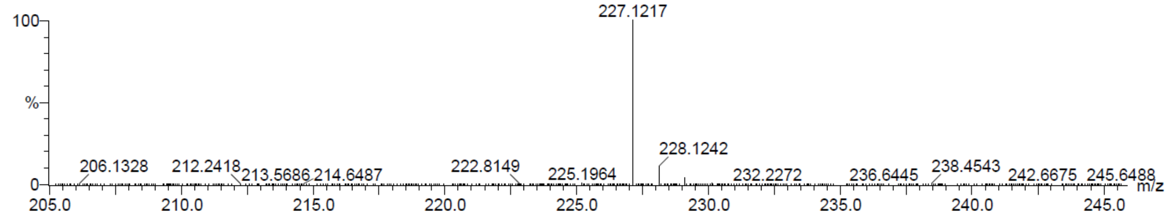

Minimum:				-1.5				
Maximum:		5.0	5.0	80.0				
Mass	Calc. Mass	mDa	PPM	DBE	i-FIT	Norm	Conf(%)	Formula
227.1217	227.1218	-0.1	-0.4	3.5	904.4	n/a	n/a	C11 H19 N2 O S

Figure S23. HRMS spectra of compound **3e**

Elemental Composition Report

3f

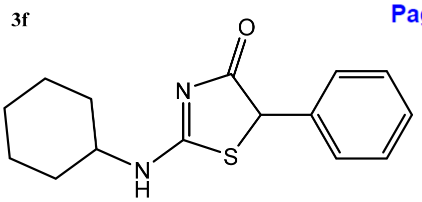

Page 1

Single Mass Analysis

Tolerance = 10.0 PPM / DBE: min = -1.5, max = 70.0
Element prediction: Off
Number of isotope peaks used for i-FIT = 9

Monoisotopic Mass, Even Electron Ions
23 formula(e) evaluated with 1 results within limits (all results (up to 1000) for each mass)
Elements Used:
C: 0-20 H: 0-25 N: 0-2 O: 0-1 S: 0-1
200526_Patryk_6A 25 (0.277) Cm (25:30-3:8)

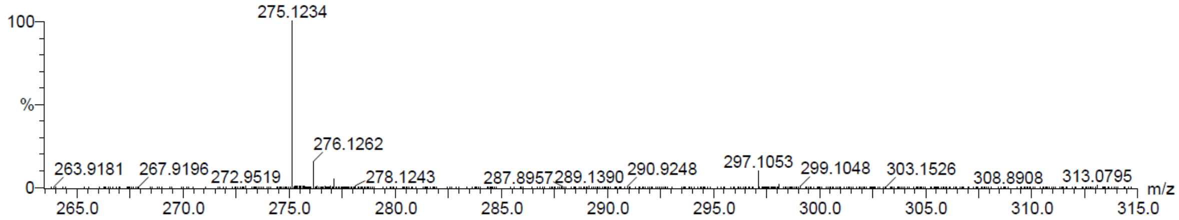

Minimum:				-1.5				
Maximum:		5.0	10.0	70.0				
Mass	Calc. Mass	mDa	PPM	DBE	i-FIT	Norm	Conf(%)	Formula
275.1234	275.1218	1.6	5.8	7.5	1341.2	n/a	n/a	C15 H19 N2 O S

Figure S24. HRMS spectra of compound **3f**

Elemental Composition Report

3g

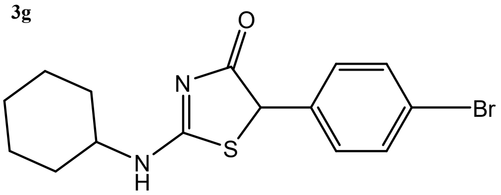

Page 1

Single Mass Analysis
Tolerance = 5.0 PPM / DBE: min = -1.5, max = 80.0
Element prediction: Off
Number of isotope peaks used for i-FIT = 9

Monoisotopic Mass, Even Electron Ions
538 formula(e) evaluated with 2 results within limits (all results (up to 1000) for each mass)
Elements Used:
C: 0-60 H: 0-70 N: 0-6 O: 0-4 S: 0-1 Br: 0-1

210318_7_1_PatrykA 18 (0.205) Cm (18:30) TOF MS ES+ 4.78e+006

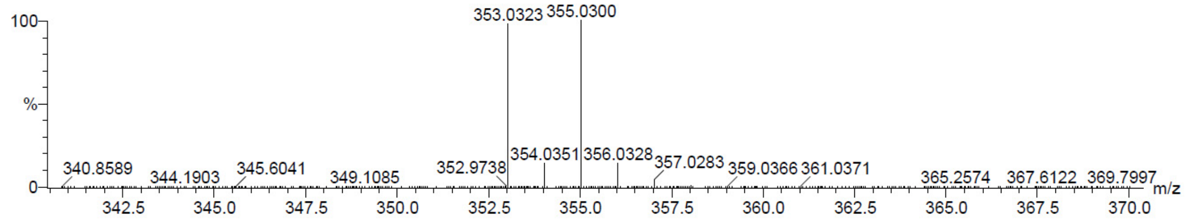

Minimum:				-1.5				
Maximum:		5.0	5.0	80.0				
Mass	Calc. Mass	mDa	PPM	DBE	i-FIT	Norm	Conf(%)	Formula
353.0323	353.0323	0.0	0.0	7.5	1182.6	0.000	100.00	C15 H18 N2 O S Br
	353.0311	1.2	3.4	19.5	1210.2	27.625	0.00	C19 H5 N4 O4

Figure S25. HRMS spectra of compound **3g**

Elemental Composition Report

3h

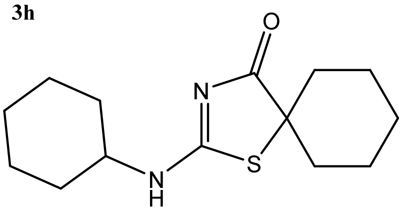

Page 1

Single Mass Analysis
Tolerance = 5.0 PPM / DBE: min = -1.5, max = 80.0
Element prediction: Off
Number of isotope peaks used for i-FIT = 9

Monoisotopic Mass, Even Electron Ions
237 formula(e) evaluated with 1 results within limits (all results (up to 1000) for each mass)
Elements Used:
C: 0-60 H: 0-70 N: 0-6 O: 0-4 S: 0-1
210318_8_1_PatrykA 28 (0.303) Cm (28:40-3:8) TOF MS ES+ 2.17e+007

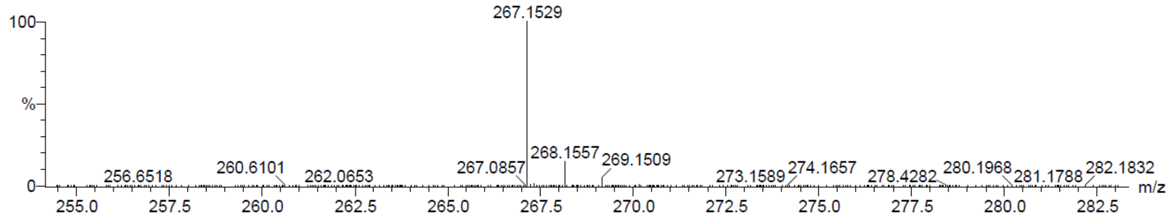

Minimum:				-1.5				
Maximum:		5.0	5.0	80.0				
Mass	Calc. Mass	mDa	PPM	DBE	i-FIT	Norm	Conf(%)	Formula
267.1529	267.1531	-0.2	-0.7	4.5	1136.1	n/a	n/a	C14 H23 N2 O S

Figure S26. HRMS spectra of compound **3h**

Elemental Composition Report

3i

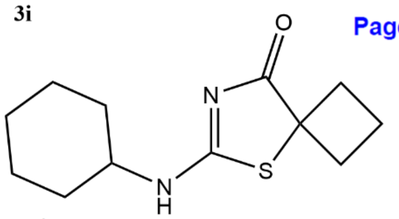

Page 1

Single Mass Analysis
Tolerance = 5.0 PPM / DBE: min = -1.5, max = 80.0
Element prediction: Off
Number of isotope peaks used for i-FIT = 9

Monoisotopic Mass, Even Electron Ions
214 formula(e) evaluated with 1 results within limits (all results (up to 1000) for each mass)
Elements Used:
C: 0-60 H: 0-70 N: 0-6 O: 0-4 S: 0-1
210318_9_1_PatrykA 29 (0.311) Cm (29:42)

TOF MS ES+
3.29e+007

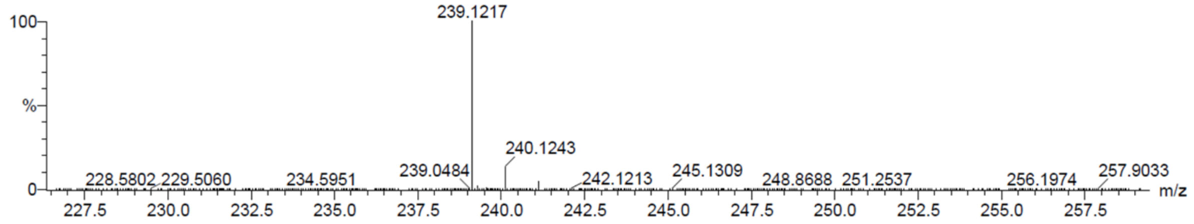

Minimum:				-1.5				
Maximum:		5.0	5.0	80.0				
Mass	Calc. Mass	mDa	PPM	DBE	i-FIT	Norm	Conf(%)	Formula
239.1217	239.1218	-0.1	-0.4	4.5	1258.7	n/a	n/a	C12 H19 N2 O S

Figure S27. HRMS spectra of compound **3i**

(C). Structural Alert Analysis

Table S1. In silico structural alerts (PAINS and Brenk) for investigated compounds.

Compound	Alerts (Yes/No)	
	PAINS	Brenk
**3a**	No	No
**3b**	No	No
**3c**	No	No
**3d**	No	No
**3e**	No	No
**3f**	No	No
**3g**	No	No
**3h**	No	No
**3i**	No	No
